# Respiratory Syncytial Virus Infections in Recipients of Bone Marrow Transplants: A Systematic Review and Meta-Analysis

**DOI:** 10.3390/idr16020026

**Published:** 2024-03-29

**Authors:** Matteo Riccò, Salvatore Parisi, Silvia Corrado, Federico Marchesi, Marco Bottazzoli, Davide Gori

**Affiliations:** 1 AUSL–IRCCS di Reggio Emilia, Servizio di Prevenzione e Sicurezza Negli Ambienti di Lavoro (SPSAL), Local Health Unit of Reggio Emilia, 42122 Reggio Emilia, Italy; 2 Sanofi, Medical Affairs, 20100 Milan, Italy; 3 ASST Rhodense, Dipartimento della donna e Area Materno-Infantile, UOC Pediatria, 20024 Milan, Italy; scorrado@asst-rhodense.it; 4 Department of Medicine and Surgery, University of Parma, 43126 Parma, Italy; 5 Department of Otorhinolaryngology, APSS Trento, 38122 Trento, Italy; 6 Department of Biomedical and Neuromotor Sciences, University of Bologna, 40126 Bologna, Italy

**Keywords:** RSV, viral pneumonia, differential diagnosis, bone marrow transplantation

## Abstract

Human Respiratory Syncytial Virus (RSV) is a common cause of respiratory tract infections. Usually associated with infants and children, an increasing amount of evidence suggests that RSV can cause substantial morbidity and mortality in immunocompromised individuals, including recipients of bone marrow transplantation (BMT). The present systematic review was therefore designed in accordance with the PRISMA guidelines to collect available evidence about RSV infections in BMT recipients. Three medical databases (PubMed, Embase, and MedRxiv) were therefore searched for eligible observational studies published up to 30 September 2023 and collected cases were pooled in a random-effects model. Heterogeneity was assessed using I^2^ statistics. Reporting bias was assessed by means of funnel plots and regression analysis. Overall, 30 studies were retrieved, including 20,067 BMT cases and 821 RSV infection episodes. Of them, 351 were lower respiratory tract infections, and a total of 78 RSV-related deaths were collected. A pooled attack rate of 5.40% (95% confidence interval [95%CI] 3.81 to 7.60) was identified, with a corresponding incidence rate of 14.77 cases per 1000 person-years (95%CI 9.43 to 20.11), and a case fatality ratio (CFR) of 7.28% (95%CI 4.94 to 10.60). Attack rates were higher in adults (8.49%, 95%CI 5.16 to 13.67) than in children (4.79%, 95%CI 3.05 to 7.45), with similar CFR (5.99%, 95%CI 2.31 to 14.63 vs. 5.85%, 95%CI 3.35 to 10.02). By assuming RSV attack rates as a reference group, influenza (RR 0.518; 95%CI 0.446 to 0.601), adenovirus (RR 0.679, 95%CI 0.553 to 0.830), and human metapneumovirus (RR 0.536, 95%CI 0.438 to 0.655) were associated with a substantially reduced risk for developing corresponding respiratory infection. Despite the heterogeneous settings and the uneven proportion of adult and pediatric cases, our study has identified high attack rates and a substantial CFR of RSV in recipients of BMT, stressing the importance of specifically tailored preventive strategies and the need for effective treatment options.

## 1. Introduction

Respiratory Syncytial Virus (RSV) is a medium-sized (120–300 nm diameter), pleomorphic, enveloped virus with a negative sense, single stranded RNA genome (15 to 16 kb) that belongs to the genus orthopneumovirus (family *Pneumoviridae*) [1,2]. RSV is a quite contagious pathogen: at community-level, it has a geographically defined seasonal trend [3,4,5] that, in the Northern hemisphere, extensively overlaps with other respiratory viruses such as influenza, adenovirus (HAdV), and SARS-CoV-2 [6,7]. Before the SARS-CoV-2 pandemic, RSV circulated in countries with temperate climates throughout the winter season, peaking between December and January [5,8], while in tropical countries RSV outbreaks were clustered during hot, humid, and rainy days of the summer season [2,9,10]. RSV has been acknowledged as the single most common viral cause of lower respiratory tract infections (LRTI), and is considered a common cause of morbidity among young children (around 33 million cases by year) [11]. Nonetheless, these figures are reasonably underestimated. On the one hand, RSV mostly causes upper respiratory tract infections (URTI) [2,4], characterized by mild respiratory symptoms [4,8,11,12], that only in a reduced proportion of cases evolve to LRTI [13]. On the other hand, nearly all children will be infected with RSV before the 24th month of age [4,14,15,16,17], and most of them are usually managed as outpatients [11], even in the case of LRTI.

RSV has often been regarded as a pediatric pathogen, as it causes high hospitalization rates in infants [18,19,20,21,22], even in healthy ones [8,23,24], but respiratory RSV infections are not limited to pediatric-age subjects [23,25,26,27], and RSV is increasingly acknowledged as a common cause of respiratory illnesses in adults [2]. RSV can cause a substantial burden of disease in all immunocompromised subjects [26,28], irrespective of their actual age: as a consequence, it has been associated with high morbidity and mortality in elderly populations [27,29,30], particularly among institutionalized subjects [8,31,32,33], but also among subjects affected by solid tumors [34,35,36], hematologic malignancies [37], and HIV/AIDS [38], with a significant public health impact [36,37,38,39,40,41].

Among immunocompromised subjects, a high-risk subgroup has previously been identified in recipients of bone marrow transplantation (BMT), as their clinical course tends to be particularly aggressive [42,43,44,45]. On the one hand, up to two thirds of RSV infections in BMT develop as LRTIs [18,46]. In turn, RSV-related LRTIs occurring in BMT recipients have been characterized by very high lethality, which in some studies ranged between 10% and 20% of all cases [47,48,49,50,51], but which could reach 70% or even 100% when antiviral therapy started after the onset of respiratory failure [18]. On the other hand, both preventive and treatment options are limited. Although in 2023 two preventive vaccines were licensed for human use (Abrysvo from Pfizer Inc. (Pfizer Europe MA EEIG, Brussels, Belgium) and Arexvy from GlaxoSmithKline LLC (GlaxoSmithKline Biologicals SA, Rixensart, Belgium)) [52,53], their overall efficacy has only been well documented in older adults [54,55], with a subsequent recommendation for individuals aged ≥60 years, and no specific assessment on recipients of BMT or SOT has been provided. Monoclonal antibodies (mAb) such as palivizumab and nirsevimab have been proven to be quite effective in preventing severe cases of RSV, particularly when dealing with RSV-related LRTI [23,56,57,58,59,60], but again no specific recommendations for BMT recipients have been issued. To date, the only available treatment option is represented by ribavirin [45,61,62,63,64,65,66,67,68,69,70]; however, it should be stressed that neither the United States Food and Drug Administration (FDA) nor the European Medicine Agency (EMA) has approved the use of ribavirin for conditions other than hepatitis C [18,44,61,67,69].

Even though several high-quality narrative reviews have specifically addressed the topic of RSV infections in BMT recipients [18,44], to the best of our knowledge no systematic review has been performed to date to summarize the available evidence on this specific topic. As an updated definition of the actual RSV burden of disease among BMT recipients is needed to inform health policies and for both preventive and treatment guidelines, a synthesis of the available literature was performed to ascertain (1) whether RSV infection may be acknowledged as a rare occurrence in BMT recipients or not, and (2) whether available evidence confirms that RSV infections in BMT recipients are associated with high lethality or not.

## 2. Materials and Methods

### 2.1. Research Concept

The present systematic review and meta-analysis of the literature was performed in accordance with the “Preferred Reporting Items for Systematic Reviews and Meta-Analysis” (PRISMA) guidelines and registered in the international database of prospectively registered systematic reviews in health and social care, welfare, public health, education, crime, justice, and international development (PROSPERO) with the progressive registration number CRD42023468469 [71,72] (PRISMA Checklist is available as Supplementary Table S1).

The preliminary step was the definition of research concepts by means of the “PECO” strategy (i.e., Patient/Population/Problem; Exposure; Control/Comparator; Outcome) [71,72], as summarized in Table 1. More precisely, the systematic review was designed to assess the occurrence of Respiratory Syncytial Virus infections (E) among individuals (children and adults) having received bone marrow transplantation (P), in order to properly define the occurrence of RSV infections, LRTI, and corresponding lethality (O). As a comparator (C), where available, data on the occurrence of other respiratory tract infections were retrieved (and particularly on Influenza, HAdV, human Metapneumovirus [hMPV], and SARS-CoV-2).

### 2.2. Research Strategy

The search strategy resulted from the combination of the following search strings:

(a) PubMed (through Medical Subject Heading [MeSH] terms): (“RSV” OR “respiratory syncytial virus, human [Mesh]” OR “bronchiolitis [Mesh]”) AND (“bone marrow transplantation [Mesh]” OR “hematopoietic stem cell transplantation [Mesh]” OR “mesenchymal stem cell transplantation [Mesh]”).
(b) EMBASE: (“bone marrow transplantation”/exp OR “bone marrow transplantation” OR “hematopoietic stem cell transplantation”) AND (“human respiratory syncytial virus” OR “respiratory syncytial virus infection” OR “respiratory syncytial virus pneumonia”).
(c) medRxiv: (“RSV” OR “respiratory syncytial virus, human” OR “bronchiolitis”) AND (“bone marrow transplantation” OR “hematopoietic stem cell transplantation” OR “mesenchymal stem cell transplantation”).

All databases were searched from inception up to 30 October 2023, without applying any reverse-chronological restrictions, in the following languages: English, Italian, German, French, Spanish, Portuguese.

### 2.3. Screening

Documents eligible for being included in the present review were original studies with a prospective or retrospective design (i.e., cohort, case–control and cross-sectional studies) on subjects having previously received BMT, irrespective of the baseline status. Case series and case reports, as well as reports on clusters of RSV infections, were not included in the pooled analyses.

Exclusion criteria were as follows:

(1) Full text not available through online repositories or through inter-library loan;
(2) Reports lacking appropriate or only vaguely defined geographical settings and corresponding timeframes;
(3) Diagnosis for RSV infection provided by means of diagnostic tests other than Real Time quantitative Polymerase Chain Reaction (RT-qPCR) or by means of clinical features of the patient(s);
(4) Reports lacking the total number of RSV infections and only providing the amount of either URTI or LRTI;
(5) Studies not including the total number (i.e., the denominator) of BMT cases from the parent institution(s) for that timeframe.

As recommended by the PRISMA statement [71,72], items were initially title-screened for their relevance to the subject, and their abstracts were subsequently analyzed. All the entries that were considered consistent with the aims of the present review were then screened by their full text in order to ascertain their consistency with the inclusion criteria. All retrieved items were independently rated by two investigators (AB, FM), and their disagreements were either resolved by consensus or through input from the chief investigator (MR) when a preliminary consensus between investigators was not reached.

### 2.4. Summary of Retrieved Data

Data abstracted included:

(a) Setting of the study: country, region, year (timeframe);
(b) Amount of BMT included in the estimates (autologous vs. allogenic);
(c) Number of cases with patients aged <18 y.o. at the time of the study (i.e., children/adolescents) vs. cases ≥ 18 y.o. (adults);
(d) Characteristics of RSV cases: total cases, number of LRTI, number of URTI, and number of RSV-related deaths;
(e) Where available, other respiratory tract viral infections (i.e., influenza, HAdV, hMPV, and SARS-CoV-2) and total number of reported cases;
(f) Total number of cases treated with palivizumab (if available).

### 2.5. Risk of Bias Analysis

The risk of bias (ROB) of retrieved studies was performed by means of the ROB tool from the National Toxicology Program (NTP)’s Office of Health Assessment and Translation (OHAT) [73,74,75]. OHAT ROB focuses on the internal validity of a given study by weighting six possible sources of bias (i.e., participant selection, confounding, attrition/exclusion, detection, selective reporting, and other sources). Rather than aiming to identify articles to be removed from the analyses, the ROB tool assesses the likelihood of any of its dimensions compromising (or not) the likelihood between exposure and outcome, with potential answers ranging from “definitely low”, “probably low”, and “probably high”, to “definitely high”. In fact, the OHAT ROB tool neither applies an overall rating for each study nor requires that studies affected by potential risk of bias (i.e., those rated with “probably high” or “definitely high” bias assessment in any of its 6 dimensions) are removed from the pooled analyses. As for the screening procedures, retrieved items were preliminarily and independently rated by two investigators (AB, FM), and disagreements were either resolved by consensus or through input from the chief investigator (MR).

### 2.6. Data Analysis

All estimates from the included studies were initially summarized through a descriptive analysis, with the subsequent calculation of attack rate estimates for RSV. Attack rates were defined as the number of people who developed viral infection out of the number of people at risk for the illness and reported as cases per 100 population. If a study did not include raw data, either as prevalent cases or a referent population, such figures were reverse-calculated from the available data. Incidence rates were calculated by a cumulative calculation of person-years observation time provided by each study. The Case Fatality Ratio (CFR) for RSV was calculated as the percentage rate of RSV-related deaths.

Risk Ratios (RR) for RSV-positive status and RSV-related deaths and their corresponding 95% Confidence Intervals (95%CI) were calculated using bivariate analysis by assuming the following arbitrary reference groups: country, USA; timeframe, 2015 onwards; group of patients, adults and pediatrics; study design, single center.

Pooled estimates were calculated through a meta-analysis of retrieved studies. A random effect model (REM) was preferred over a fixed effect model (FEM) because of the presumptive heterogeneity of retrieved studies in terms of sample size, design, and eventual identification of reported outcome [76,77]. The inconsistency in the effect between included studies was defined as the percentage of total variation across studies likely due to heterogeneity rather than chance [75], and was quantified by the calculation of the I^2^ statistic and corresponding 95% confidence intervals (95%CI). I^2^ estimates were classified as follows: 0 to 25%, low heterogeneity; 26% to 40%, moderate heterogeneity; ≥40%, substantial heterogeneity. Confidence intervals of I^2^ estimates were provided in order to cope with the potential small size of the meta-analyses [75].

A sensitivity analysis was performed to evaluate the effect of each study on the pooled estimates by the exclusion of one study at a time. Any significant change in pooled estimates was reported. Potential publication bias was ascertained through the calculation of contour-enhanced funnel plots, and their asymmetry was eventually assessed by means of the Egger test statistic. Small study bias was eventually assessed by generating corresponding radial plots.

All calculations were performed in R (version 4.3.1) [78] and Rstudio (version 2023.06.0 Build 421; Rstudio, PBC; Boston, MA, USA) software by means of the packages meta (version 6.5-0) and fmsb (version 0.7.5). The package meta provides standard methods for meta-analysis, while the package fmsb provides functions for medical statistics and the handling of demographic data. The Prisma2020 flow diagram was designed by means of the PRISMA2020 package [79].

## 3. Results

### 3.1. Descriptive Analysis

**Figure 1:**
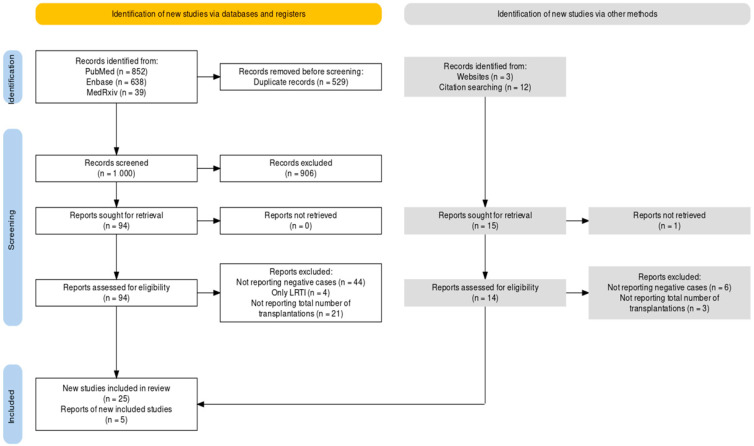
Flowchart of included studies.

As shown in Figure 1, a total of 1529 entries were pooled from database searches, including 852 entries from PubMed (55.72%), 683 from Embase (44.67%), and 39 from MedRxiv (2.55%). After the removal of duplicated records (34.60% of the original sample), a total of 1000 records were title- and abstract-screened; of them, 906 records were removed because they were not consistent with the aims and inclusion/exclusion criteria of the study (59.25% of the original sample). The remaining 94 entries were sought for retrieval (6.15% of the original sample), and then assessed for their eligibility. Of them, 44 were removed from the analyses because of the lack of estimates on RSV-negative cases (2.88%), while 21 studies were removed due to not reporting the total number of BMT cases included in the analyses (1.37%). Finally, four studies (0.26%) were not included in the qualitative and quantitative analyses as they only reported estimates on RSV-related LRTI, with no data on the total number of RSV infections.

Citation searching from the eventual sample of 25 papers obtained through database searches (1.64% of the initial sample) identified 16 potential additional records. Of them, one was not retrieved, while nine out of the remaining fourteen studies assessed for eligibility were excluded as not reporting the overall number of negative cases, or the total number of BMT (six and three entries, respectively).

Qualitative and quantitative analyses were therefore performed on a final pool of 30 papers, whose content is summarized in Table 2 [46,51,61,68,70,80,81,82,83,84,85,86,87,88,89,90,91,92,93,94,95,96,97,98,99,100,101,102,103,104], all of them published from 1999 to 2022, and including series from 1989 to 2021. More precisely, seven studies were published before 2005 [51,70,84,86,91,94,101], thirteen between 2005 and 2014 [46,68,80,82,88,89,90,92,93,95,97,100,104], and seven between 2015 and 2019 [61,81,83,87,98,102,103], while only three studies had been published since 2020 [85,96,99]. The pooled sample encompassed a total of 20,067 BMT cases (130,622.81 person-years), 821 cases of RSV infections (crude incidence rate 4.09%, and 6.285 per 1000 person-years), and 78 RSV-related deaths (crude mortality, 3.887 deaths per 1000 BMT cases and 0.597 deaths per 1000 person-years; crude CFR 9.50%) [46,51,61,68,70,80,81,82,83,84,85,86,87,88,89,90,91,92,93,94,95,96,97,98,99,100,101,102,103,104].

The largest share of reports (11 out of 30, 36.67%) was obtained from the United States [61,81,83,84,86,89,92,98,100,101,104], and included more than half of total BMT cases (12,777; 63.67%). Three further studies reported on BMT cases from the UK [51,94,97] (7.32% of total BMT cases), and there were two studies each from Canada [46,87] (6.50% of total BMT cases), Germany [68,70], South Korea [82,90], and Switzerland [88,96] (1.81%, 0.87%, and 2.78% of total BMT cases, respectively). The remaining reports were from mainland China [99,103], India [79], Italy [95], Mexico [85], Singapore [102], Spain [93], and Sweden [80], with a multi-country study [91] on 37 BMT centers and 1972 cases (9.83% of total cases) across Europe.

Most available reports were based on a single center with a retrospective design, with only three multicenter reports (for a total of 5055 BMT cases, 25.19% of the total sample) [83,91,98] and five prospective studies (660 BMT cases, 3.29%) [85,89,95,96,99]. Among the collected studies, 24 provided the proportion of allogenic BMT (65.21% of the total sample), while this information was not included in 6 studies for a total of 7047 BMT cases [84,89,92,97,98,99] (Table 3). Similarly, the proportion of pediatric cases was provided by 22 studies [46,51,61,68,81,82,83,85,86,87,90,92,93,94,95,96,98,99,101,102,103,104], for a total of 5936 subjects (29.58% of the total sample).

Overall, 22 samples included either children or adults and children/adolescents [61,70,80,81,82,83,85,87,89,90,91,92,94,97,98,100,101,102], for a total of 14,729 cases, but 6 studies did not provide the actual number of pediatric cases [70,80,89,91,97,100]. The median observation time was 51.2 months [76,93], ranging from around 3 months [68] to over 161 [92]. A total of 130,622.81 person-years was therefore collected, with a median value of 1878.32 person-years [46,81] (range from 16.83 person-years [68] to 42,838.33 person-years [84]). By considering studies performed on children alone as the reference group (4640 person-years ± 4120), the average observation time was similar in children and adults (4101 person-years ± 5265; Kruskal–Wallis test for multiple comparisons *p* = 0.999) and adults alone (4374 person-years ± 12,143; *p* = 0.078).

A total of 821 cases of RSV infections were collected, with each study including between 4 [96] and 171 cases [84], and corresponding attack rates ranging from 0.74% [97] to 39.71% [103]. The crude estimate for the whole of the sample was 8.31% ± 9.19; the estimate for children alone was not significantly different from that in adults alone (12.11% ± 12.31; Kruskal–Wallis test for multiple comparisons *p* = 0.664), or in adults and children as well (5.57% ± 6.62; *p* = 0.649). Overall, 15 studies (10,015 BMT cases) provided estimates for influenza virus detection rate (i.e., 213 cases; crude attack rate of 2.13%) [51,81,82,83,85,87,89,91,92,93,95,96,97,100,102], 12 studies reported on 102 episodes of HAdV infection from 3678 BMT cases (crude attack rate of 2.77%) [70,81,82,85,87,89,90,92,93,95,96,102], and 10 on hMPV infections, for a total of 105 episodes over 4792 BMT cases (2.19%) [81,82,83,85,87,89,93,96,97,102]. Unfortunately, the studies retrieved did not report data on the occurrence of SARS-CoV-2 infections in BMT cases.

**Figure A1:**
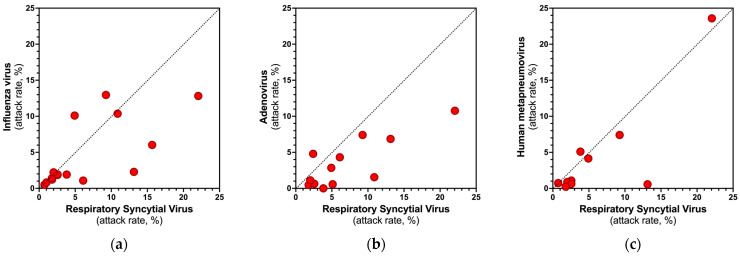
Correlation between attack rates for respiratory syncytial virus (RSV) and influenza virus (**a**), adenovirus (**b**), and human metapneumovirus (hMPV) (**c**). The correlation was significant for RSV and influenza virus (Spearman’s rho = 0.790, 95%, *p* < 0.001) and RSV and adenovirus (rho = 0.650, *p* = 0.026), but not for RSV and hMPV (rho = 0.559, *p* = 0.098) [46,51,61,68,70,80,81,82,83,84,85,86,87,88,89,90,91,92,93,94,95,96,97,98,99,100,101,102,103,104].

In studies providing estimates for other respiratory pathogens, attack rates ranged from 0.49% [97] to 12.96% [85] for influenza, from 0.0% [89] to 10.77% [85] for HAdV, and from 0.24% [87] to 23.59% [102] for hMPV. Individual attack rates for RSV were well correlated with those for influenza (Spearman’s rho = 0.790, *p* < 0.001) and HAdV infection (rho = 0.650, *p* = 0.026), while no correlation was found with hMPV (rho = 0.559, *p* = 0.098) (Appendix A, Figure A1). Individual attack rates were similar in RSV compared to influenza (Mann–Whitney [M-W] U = 84, *p* = 0.245), AdV (M-W U = 39, *p* = 0.060), and hMPV (M-W U = 32.50, *p* = 0.197). On the other hand, by assuming RSV attack rates as a reference group, flu (RR 0.518; 95%CI 0.446 to 0.601), HAdV (RR 0.679, 95%CI 0.553 to 0.830), and hMTP (RR 0.536, 95%CI 0.438 to 0.655) were characterized by a substantially reduced risk for developing corresponding respiratory infection.

As shown in Table 4, the risk for developing RSV infection was lower in multicenter studies compared to single center ones (RR 0.445, 95%CI 0.364 to 0.544), in studies performed in adults only (RR 1.846, 95%CI 1.580 to 2.162) and in pediatric patients only (RR 1.264, 95%CI 1.054 to 1.517) than in studies including both children and adults.

By considering studies performed from 2015 onwards as the reference group, the risk for developing RSV infection was higher in the timeframe 2010–2014 (RR 1.665, 95%CI 1.366 to 2.029) and lower in the timeframe 2005–2009 (RR 0.682, 95%CI 0.553 to 0.841), with no significant differences for the studies performed before 2005 (RR 0.867, 95%CI 0.733 to 1.025). The risk for RSV infection was highest in studies from China (RR 10.784, 95%CI 7.915 to 14.596), followed by Singapore (RR 5.959, 95%CI 4.520 to 7.885), South Korea (RR 3.558, 95%CI 2.406 to 5.260), Sweden (RR 3.150, 95%CI 2.248 to 4.414), India (RR 2.978, 95%CI 1.693 to 5.236), Italy (RR 2.945, 95%CI 1.948 to 4.453), Mexico (RR 2.506, 95%CI 1.082 to 5.804), and Switzerland (RR 1.504, 95%CI 1.056 to 2.141), while the single multi-center study from European countries was associated with the lowest risk (RR 0.274, 95%CI 0.176 to 0.428).

**Figure A2:**
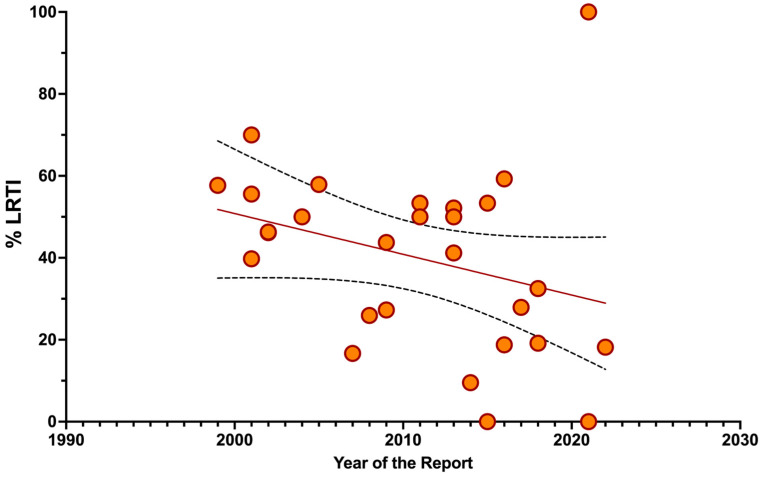
Time trend of the proportion of LRTI over the total number of RSV cases. A non-significant negative association was eventually identified (rho = −0.366; *p* = 0.055) [46,51,61,68,70,80,81,82,83,84,85,86,87,88,89,90,91,92,93,94,95,96,97,98,99,100,101,102,103,104].

**Figure A3:**
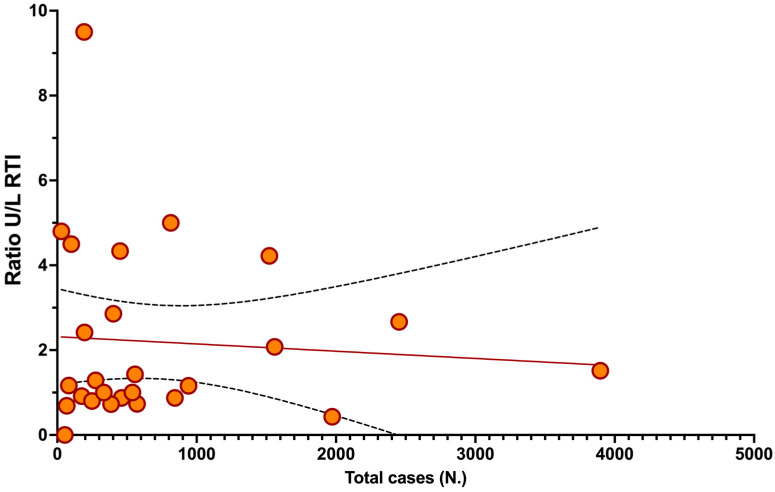
Correlation between sample size and the ratio between upper and lower respiratory tract infections (URTI; LRTI) occurring in 20,067 cases of bone marrow transplantation collected in the present survey. No actual association was found (rho = 0.833; *p* = 0.724) [46,51,61,68,70,80,81,82,83,84,85,86,87,88,89,90,91,92,93,94,95,96,97,98,99,100,101,102,103,104].

Nearly all studies provided the proportion of LRTI over the total cases of RSV infections [46,51,61,68,70,80,81,82,83,84,85,86,87,88,91,92,93,94,95,96,97,98,99,100,101,102,103,104], with the notable exception of the reports from Kuypers et al. [89] and from Lee et al. [90]; that is, a total of 333 BMT cases (1.66% of total cases), and 15 out 821 RSV cases (1.83% of total RSV cases). Overall, 315 LRTI cases were reported (38.37% of total RSV cases), with a proportion ranging from 0 [81,96] to 100% [96]. The proportion of LRTI over total cases decreased over time, but no significant time-trend was identified (rho = −0.366; *p* = 0.055; Appendix A, Figure A2). Similarly, no significant correlation was reported between the ratio of LRTI over URTI and sample size (rho = 0.833; *p* = 0.724; Appendix A Figure A3). The proportion of LRTI episodes over total cases was not significantly greater in studies only performed on children (45.30% ± 27.91) than in studies only performed on adults (38.80% ± 20.30, *p* = 0.999) and in adults and children (37.73% ± 21.72, *p* = 0.999). On the other hand, data on the delivery of palivizumab were provided by only four studies, providing a total of 30 episodes (3.65% of all RSV cases), more precisely the reports of McCoy et al. [104] (50.00% of palivizumab cases), Lo et al. [92] (20.59%), Rowan et al. [98] (10.64%), and El-Bietar et al. [61] (15.63%).

A total of 78 deaths were ultimately reported. In 9 studies, all patients allegedly recovered from RSV infection [51,86,89,95,96,97,99,100,104], while in the remaining 21 studies the total number of reported cases ranged from 1 [61,81,85,87,90,92,98,103] to 30 [84]. The corresponding CFR was 8.39% ± 8.29 (range 0 to 30.00%), with no substantial differences between children and adolescents (8.09% ± 5.90) and adults (7.18% ± 9.60; *p* = 0.936) and studies containing both children and adults (10.08% ± 8.75; *p* = 0.999). As shown in Table 5, the risk for RSV-related death was similar in multicenter vs. single center studies, and by characteristics of patients. On the other hand, studies performed before 2005 had a greater risk for reporting RSV-related deaths than most recent ones (RR 2.378, 95%CI 1.333 to 4.23). When dealing with the country in which the studies were performed, by considering the United States as the reference group, an increased risk was associated with Germany alone (RR 3.198, 95%CI 1.436 to 7.119), while all other countries reported similar estimates.

**Figure A4:**
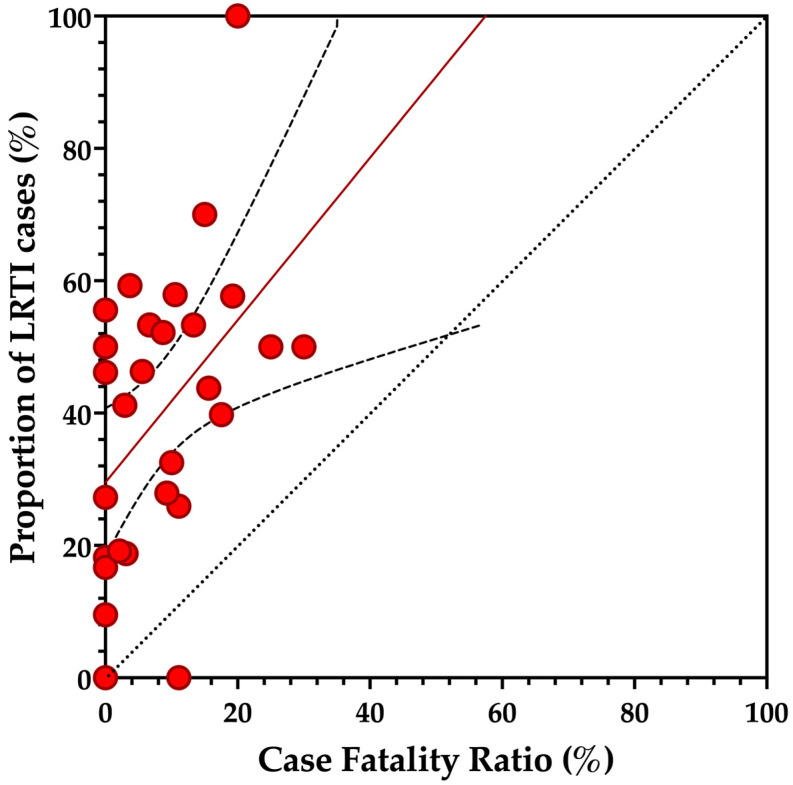
Correlation of case fatality ratio and proportion of lower respiratory tract infections (LRTI) in sampled cases of bone marrow transplantation [46,51,61,68,70,80,81,82,83,84,85,86,87,88,89,90,91,92,93,94,95,96,97,98,99,100,101,102,103,104]. A positive correlation was eventually found (Spearman’s rho = 0.445, *p* = 0.018).

While no significant time trend in CFR was ultimately identified (Appendix A, Figure A3), and attack rates and CFR were not correlated (Spearman’s rho = 0.099, 95%CI −0.281 to 0.452, *p* = 0.603), a positive correlation was found between the proportion of LRTI and CFR (rho = 0.445, *p* = 0.018; Appendix A Figure A4).

As shown in Table 6, the proportion of allogenic BMT cases over the total of BMT recipients was not significantly correlated with attack rate (rho = 0.311, *p* = 0.139), incidence rate (rho = −0.042, *p* = 0.846), proportion of LRTI (rho = −0.235, *p* = 0.280), and CFR (rho = 0.085, *p* = 0.693). Similarly, the proportion of pediatric cases (i.e., subjects aged less than 18 years at the time of the BMT) was not significantly correlated with attack rate (rho = 0.283, *p* = 0.201), proportion of LRTI (rho = −0.008, *p* = 0.974), and CFR (rho = 0.317, *p* = 0.150), while it was negatively correlated with incidence rate (rho = −0.433, *p* = 0.044).

### 3.2. Risk of Bias

**Figure 2:**
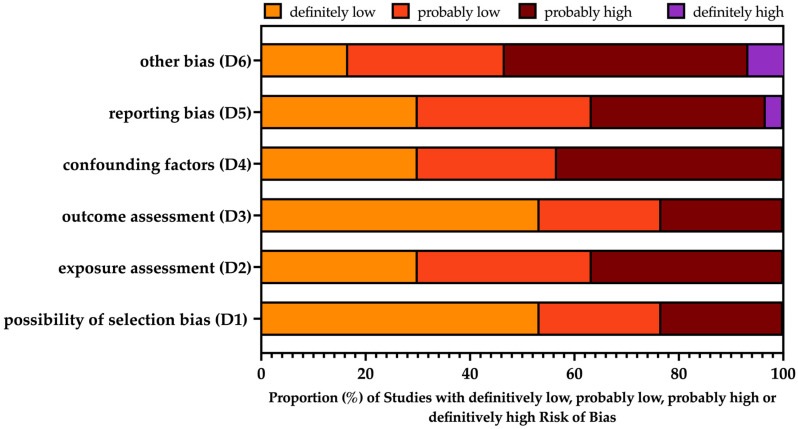
Summary of risk of bias assessment for included studies [46,51,61,68,70,80,81,82,83,84,85,86,87,88,89,90,91,92,93,94,95,96,97,98,99,100,101,102,103,104], performed according to the National Toxicology Program (NTP)’s Office of Health Assessment and Translation (OHAT) handbook and respective risk of bias (ROB) tool [74,75].

A summary of the risk of bias (ROB) assessment on retrieved studies is reported in Figure 2, while details on single studies are included in Appendix A, Table A1. Briefly, the overall quality of the collected sample was relatively high, with four studies rated as high-quality reports [83,87,93,94]. However, no study was associated with a definitively high risk of bias for the domains of selection bias (D1), exposure assessment (D2), outcome assessment (D3), and confounding factors (D4). When dealing with reporting bias, only one study was reasonably associated with a high degree of reporting bias (D5) [85], and two studies were affected by definitively high risk for other bias (D6) [85,99], as the reports were affected by the unclear reporting of individual data, including the characteristics of the BMT (i.e., autologous vs. allogenic). However, a substantial share of studies was affected by a probable high risk of bias in all domains.

As reported in Appendix A, Table A2, the most frequently reported issues were represented by the unclear reporting of demographic data, as well as of data on non-RSV cases. Moreover, whether the delivery of palivizumab was performed or at least considered as a treatment option or not was not clearly reported in a large share of reports.

### 3.3. Meta-Analysis

As shown in Table 7, the pooled attack rate for RSV was 5.40 per 100 patients (95%CI 3.81 to 7.60), with a pooled attack rate of 1.90 per 100 patients (95%CI 1.20 to 2.99) for LRTI alone.

Where available, pooled attack rates for influenza (2.65 per 100 patients; 95%CI 1.53 to 4.54), HAdV (2.10 per 100 patients, 95%CI 1.06 to 4.14), and hMPV (1.77, 95%CI 0.70 to 4.49) were similarly calculated. All estimates were affected by substantial heterogeneity (I^2^ > 60%), with estimates for RSV (94.4%, 95%CI 93.0 to 95.6), RSV-related URTI (93.0%, 95%CI 91.0 to 94.6), influenza (94.3%, 95%CI 92.1 to 95.9), and hMPV (95.8%, 95%CI 93.9 to 97.1) also exceeding 90%.

**Figure 3:**
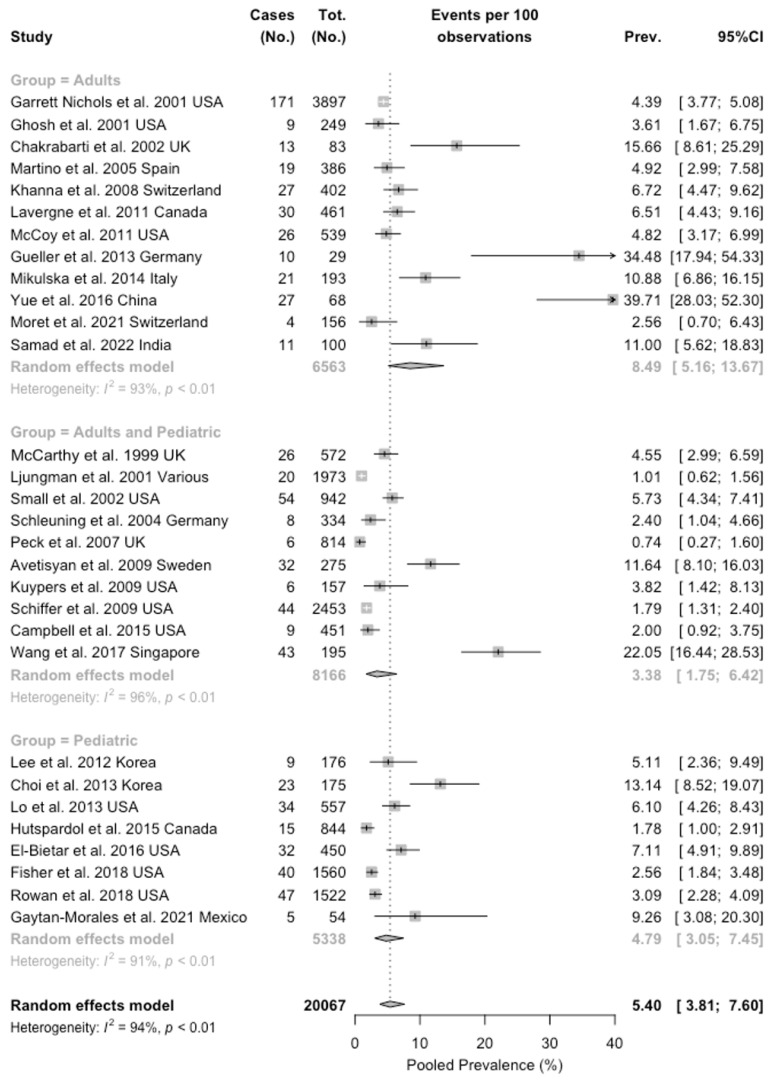
Forest plot for RSV attack rates among 20,067 subjects having undergone a bone marrow transplantation procedure. An overall estimate of 5.40% (95% confidence interval [95%CI] 3.81 to 7.60) was identified, with high heterogeneity (94.4%, 95%CI 93.0 to 95.6; *p* < 0.001) [46,51,61,68,70,80,81,82,83,84,85,86,87,88,89,90,91,92,93,94,95,96,97,98,99,100,101,102,103,104].

As shown in Figure 3, subgroup analysis brought an attack rate of 8.49 per 100 patients (5.16 to 13.67) for adults, with substantial heterogeneity (I^2^ = 93%), 4.79 per 100 patients in pediatric patients (I^2^ = 91%), and 3.38 per 100 patients in studies including both adults and children.

Overall, when taking into account the observation time (Table 8), RSV incidence was estimated at 14.77 per 1000 person-years (95%CI 9.43 to 20.12), and 5.31 per 1000 person-years for RSV-related URTI and 3.99 per 1000 person-years for RSV-related LRTI. Incidence for influenza was estimated to be 10.45, 95%CI 4.04 to 16.86, with 9.64 per 1000 person-years (95%CI 2.95 to 16.32) for HAdV infections and 15.56 per 1000 persons-years (95%CI 0.00 to 33.93) for hMPV. Heterogeneity was substantial in all analyses, particularly when dealing with RSV (I^2^ 91.6%, 95%CI 89.1 to 93.5) and hMPV (I^2^ 90.5%, 95%CI 84.6 to 94.1).

**Figure 4:**
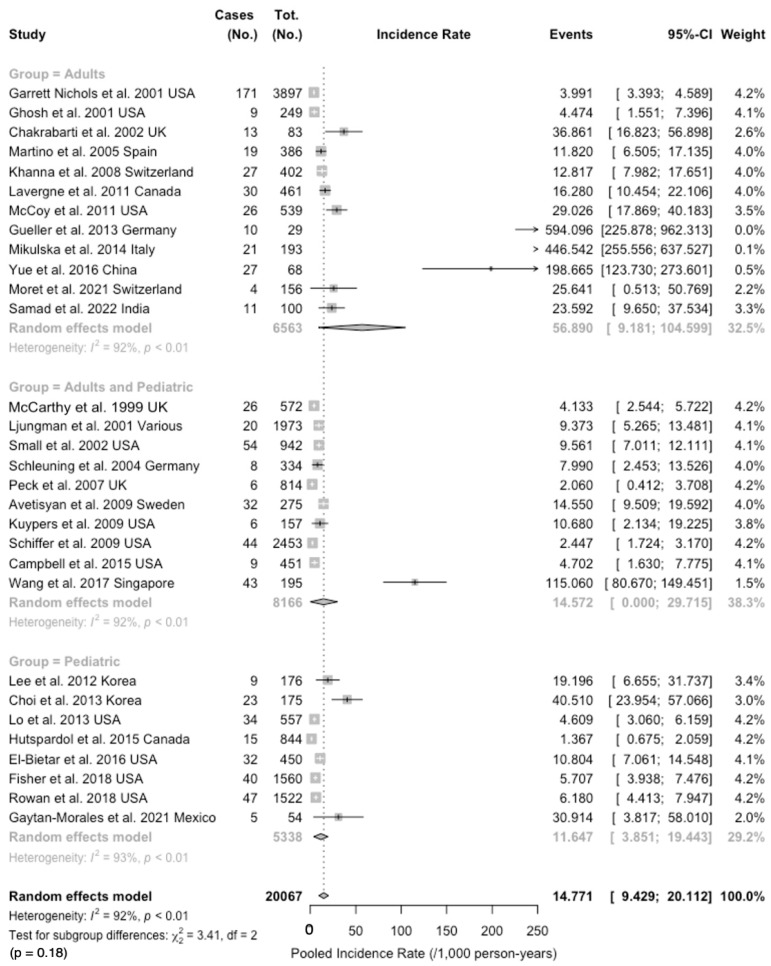
Forest plot for RSV incidence among 20,067 subjects having received a bone marrow transplantation procedure (pooled observation time: 130,622.81 person-years). An overall incidence of 14.77 cases per 1000 person-years (95% confidence interval [95%CI] 9.43 to 20.11) was identified, with high heterogeneity (91.6%, 95%CI 89.1; 93.5; *p* < 0.001) [46,51,61,68,70,80,81,82,83,84,85,86,87,88,89,90,91,92,93,94,95,96,97,98,99,100,101,102,103,104].

The subgroup analysis for incidence rates is calculated in Figure 4. In fact, incidence was highest in studies only including adults (56.89 per 1000 person-years, 95%CI 9.18 to 104.60), followed by studies including both children and adults (14.57 per 1000 person-years, 95%CI 0.00 to 29.72), and the lowest estimates were found in children alone (11.65 per 1000 person-years, 95%CI 3.85 to 19.44), with no substantial difference in tests for subgroup difference (chi squared 3.41, *p* = 0.178). Again, heterogeneity was substantial, with I^2^ estimates of 92% for studies on adults and children and 93% for studies only performed on children.

**Figure 5:**
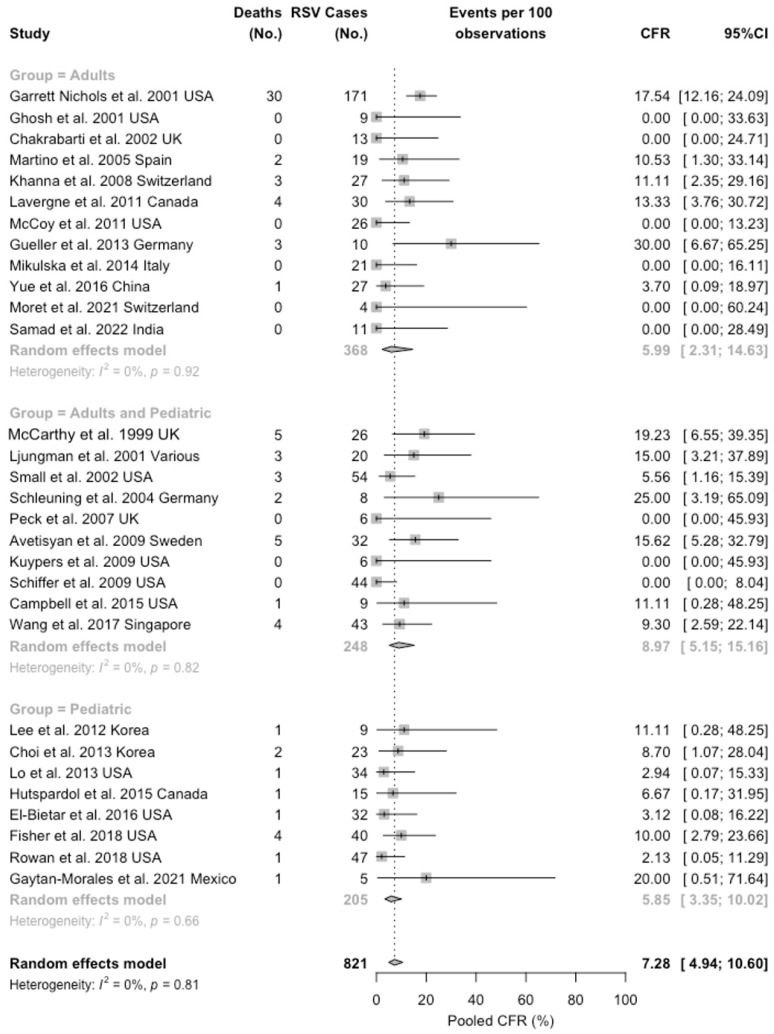
Forest plot for case fatality ratio (CFR) among 821 cases of RSV infections in people having undergone a bone marrow transplantation procedure. A total of 78 deaths were reported, for a pooled CFR of 7.28% (95% confidence interval [95%CI] 4.94 to 10.60) with low heterogeneity (I^2^ = 0.0, 95%CI 0.0 to 40.8; τ^2^ = 0.389, Q = 22.26, *p* = 0.809) [46,51,61,68,70,80,81,82,83,84,85,86,87,88,89,90,91,92,93,94,95,96,97,98,99,100,101,102,103,104].

Focusing on the CFR (Figure 5), the pooled estimate was 7.28% (95%CI 4.94 to 10.60), with the highest estimate in studies performed in both children and adults (8.97%, 95%CI 5.15 to 15.16), and similar estimates for adults (5.99%, 95%CI 2.31 to 14.63) and children (5.85%, 95%CI 3.35 to 15.16). Heterogeneity was considered low, both in general (I^2^ 0.0%, 95%CI 0.0 to 40.8) and by subgroup (all subgroup, I^2^ = 0.0%).

**Figure 6:**
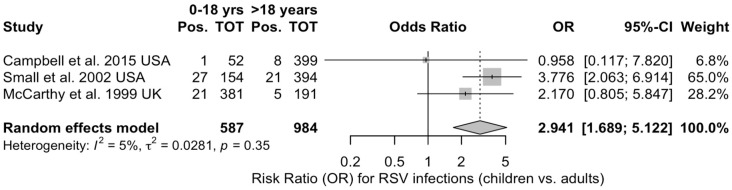
Forest plot for the odds ratio (OR) of the occurrence of Respiratory Syncytial Virus (RSV) infections in children (individuals aged 0 to 18 years) vs. adults (individuals older than 18 years). Ultimately, an OR of 2.941 (95%CI 1.689 to 5.122) was identified, with an I^2^ value of 4.8%, 95%CI 0.0 to 90.1 [81,94,101].

As only three studies provided both adult and children data, the OR for the occurrence of RSV in children vs. adults (i.e., individuals aged <18 years vs. aged 18 years or older) was calculated from this smaller subset including a total of 1571 cases (62.6% aged more than 18 years) [81,94,101]. Overall, children were associated with increased odds for developing RSV infection after BMT (OR 2.941, 95%CI 1.689 to 5.122) (Figure 6). Even though the analyses were associated with seemingly reduced heterogeneity (I^2^ = 4.8%), the corresponding 95%CI hinted at a more precautionary approach (0.0% to 90.1%).

### 3.4. Sensitivity Analysis

**Figure A5:**
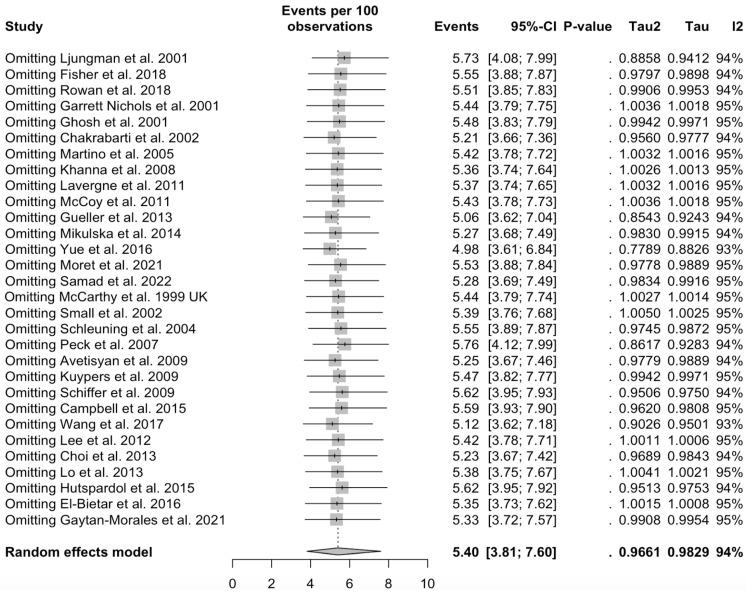
Sensitivity analysis for pooled attack rates of RSV [46,51,61,68,70,80,81,82,83,84,85,86,87,88,89,90,91,92,93,94,95,96,97,98,99,100,101,102,103,104].

**Figure A6:**
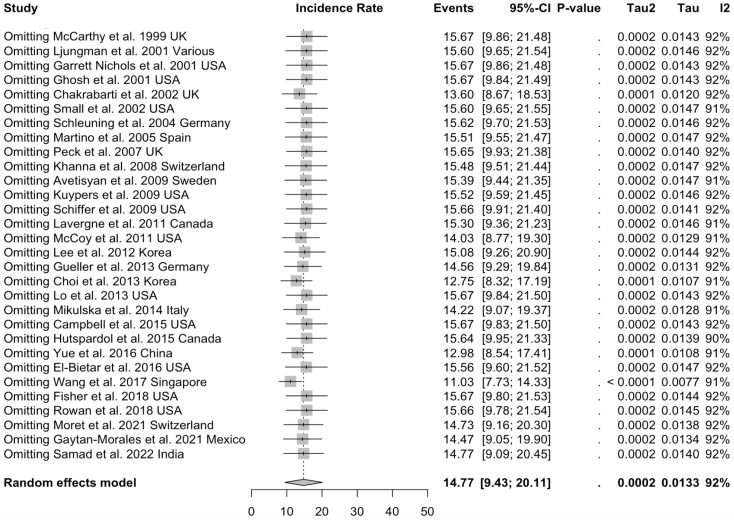
Sensitivity analysis for pooled incidence rate for RSV cases [46,51,61,68,70,80,81,82,83,84,85,86,87,88,89,90,91,92,93,94,95,96,97,98,99,100,101,102,103,104].

A sensitivity analysis was performed by removing a single study at a time. Pooled estimates for RSV attack rates (Appendix A, Figure A5) and incidence rates (Appendix A, Figure A6) were not affected in terms of residual heterogeneity, which remained consistently >90% in all analyses for attack rates and incidence rates, and around 0% for CFR. Focusing on the attack rate, estimates ranged between a minimum of 4.98% (95%CI 3.61 to 6.84), obtained through the removal of the study of Yue et al. [103], and a maximum of 5.73% (95%CI 4.08 to 7.99), obtained through the removal of the study of Ljungman et al. [91]. When dealing with incidence rates, the removal of the report by Wang et al. [102] led to the lowest estimate (11.03 cases per 1000 person years, 95%CI 7.73 to 14.33), followed by that of Choi et al. [82] (12.75 cases per 1000 person years, 95%CI 8.32 to 17.19), whilst a quite similar estimate for an attack rate of around 15.67 cases per 1000 person years was obtained by the removal of the studies of McCarthy et al. [94] (95%CI 9.86 to 21.48), Garrett Nichols et al. [84] (95%CI 9.86 to 21.48), Ghosh et al. [86] (95%CI 9.84 to 21.49), Lo et al. [92] (9.84 to 21.50), Campbell et al. [81] (95%CI 9.83 to 21.50), and Fisher et al. [83] (95%CI 9.80 to 21.53).

**Figure A7:**
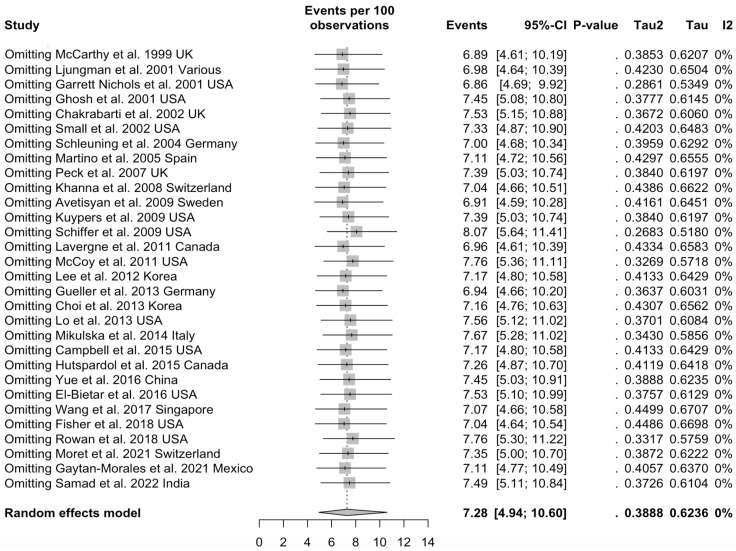
Sensitivity analysis for pooled case fatality ratio (CFR) [46,51,61,68,70,80,81,82,83,84,85,86,87,88,89,90,91,92,93,94,95,96,97,98,99,100,101,102,103,104].

CFR was similarly not affected by the removal of individual cases in terms of heterogeneity, and the lowest estimate (6.86%, 95%CI 4.69 to 9.92) was obtained by the removal of the study by Garrett Nichols et al. [84], followed by the removal of the study by McCarthy et al. [94] (6.89%, 95%CI 4.61 to 10.19), while the highest estimate resulted from the removal of the study by Schiffer et al. [100] (8.07%, 95%CI 5.64 to 11.41) (Appendix A, Figure A7).

**Figure A8:**
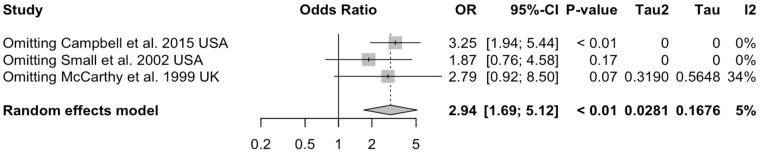
Sensitivity analysis for pooled odds ratio (OR) [81,94,101].

Eventually, the removal of the study by McCarthy et al. [94] from the pooled estimate on OR led to a noticeable change in both the pooled estimates for the occurrence of RSV infections in children vs. adults (OR 2.79, 95%CI 0.92 to 8.50) and in those for I^2^ (34%), while the removal of the study from Small et al. [101] only affected the estimates for OR and 95%CI (OR 1.87, 95%CI 0.76 to 4.58) (Appendix A, Figure A8).

### 3.5. Analysis of Publication Bias and Small Study Bias

**Figure 7:**
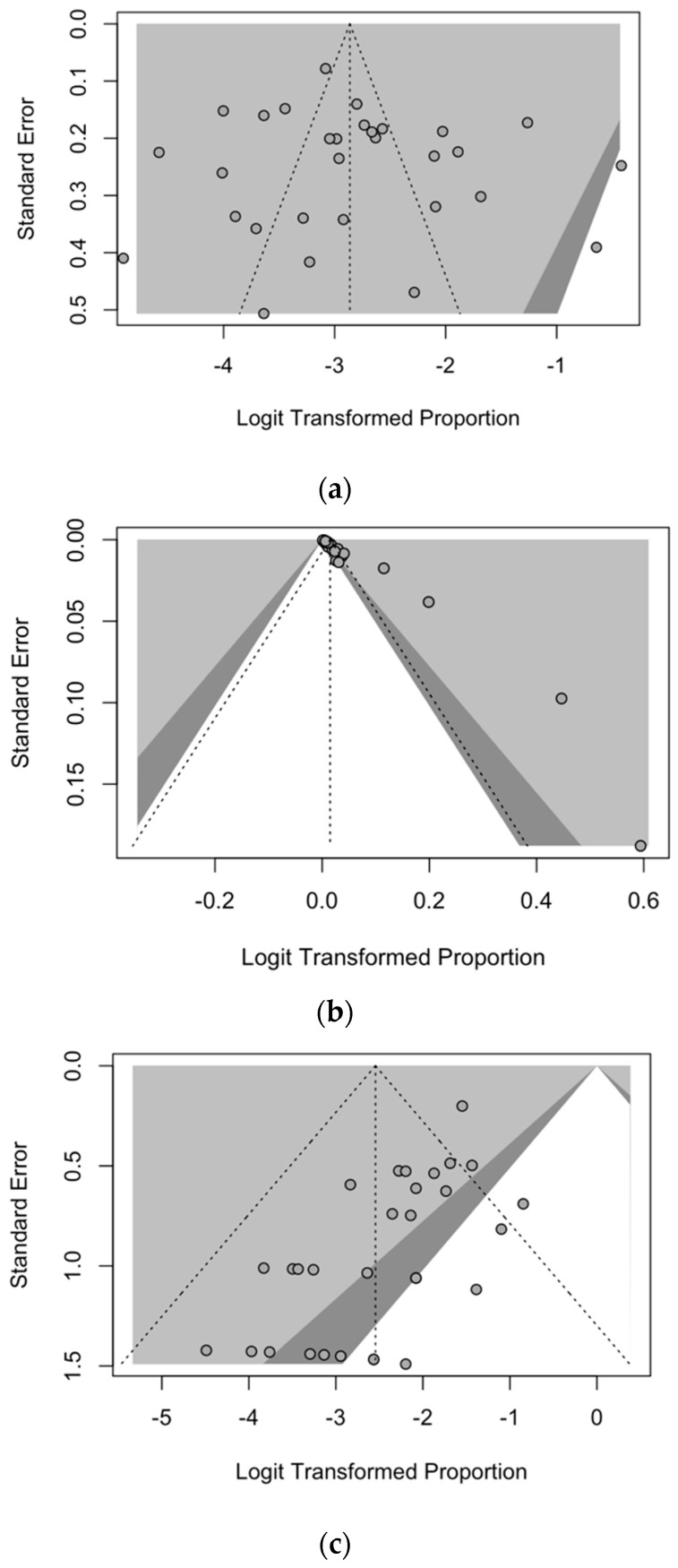
Funnels plots for the whole of the studies reported in the present meta-analysis in terms of attack rates (**a**), incidence rates for RSV (**b**), and their corresponding CFR (**c**).

According to the recommendation from PRISMA guidelines, publication bias was initially ascertained (Figure 7) by the calculation of funnel plots for attack rates (Figure 7a), incidence rates (Figure 7b), and CFR (Figure 7c). In funnel plots, the sample size is plotted against the effect size reported: as the size of the sample increases, individual estimates of the effect are likely to converge around the true underlying estimate [71,72,79]. Therefore, if the estimates are not affected by some degree of publication bias, point estimates are expected to be evenly scattered. On the other hand, if any publication bias has occurred, some asymmetry in the scatter plot in small studies can be spotted, with more studies showing a positive result than those showing a negative one.

In our study, a visual inspection of contour-enhanced funnel plots suggested that publication bias could be ascertained for incidence rates, attack rates, and CFR, as estimates were not evenly scattered across the logit of transformed proportion.

**Figure 8:**
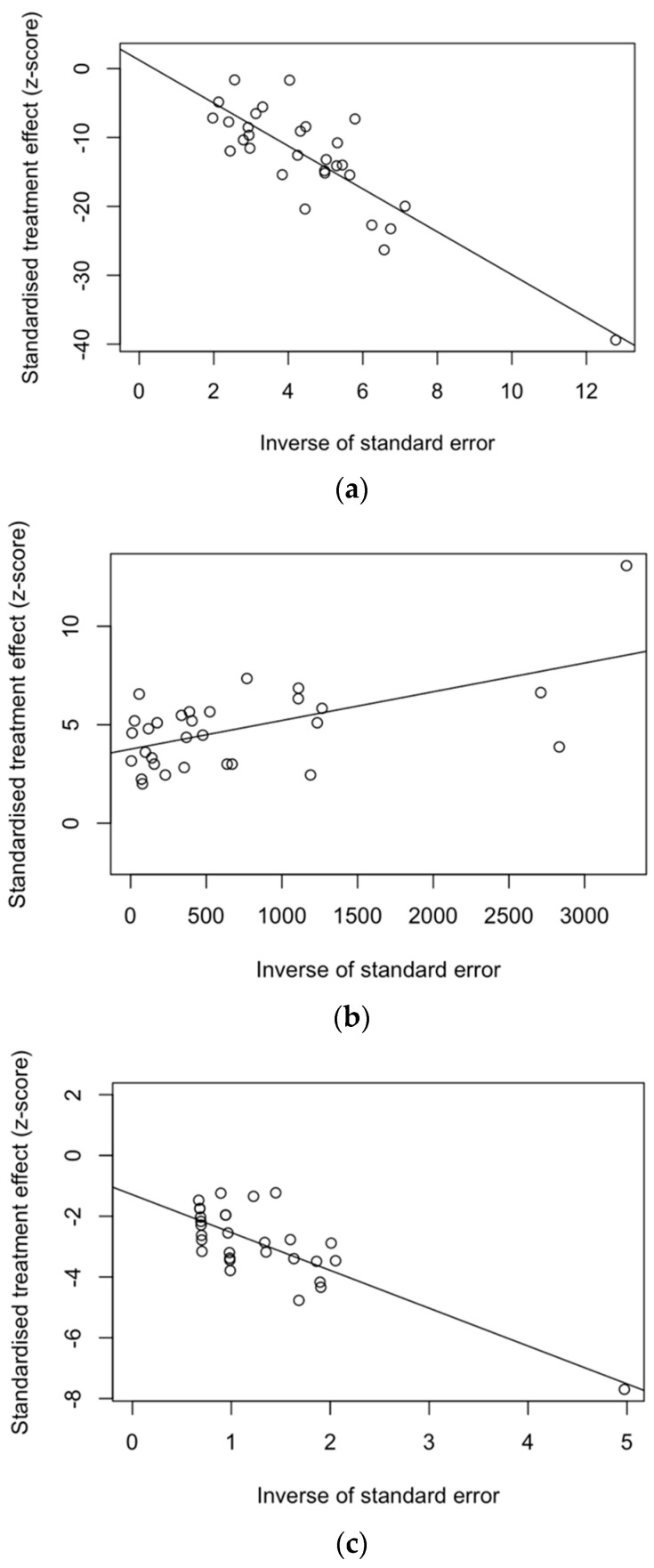
Radial plots for included studies, assessed by the calculation of attack rates (**a**); Egger’s test: t = 0.65, df = 28, *p*-value = 0.519), incidence rates (**b**); t = 8.76, df = 28, *p*-value < 0.001), and case fatality ratio (**c**); t = −4.78, df = 28, *p*-value < 0.001) [46,51,61,68,70,80,81,82,83,84,85,86,87,88,89,90,91,92,93,94,95,96,97,98,99,100,101,102,103,104].

On the other hand, point estimates were evenly scattered on both sides of regression lines in radial plots. However, Egger’s test (i.e., the linear regression analysis of the intervention effect estimates on their standard errors weighted by their inverse variance) hinted at substantial publication bias for both incidence rates (Figure 8b; t = 8.76, df = 28, *p*-value < 0.001) and CFR (Figure 8c; t = −4.78, df = 28, *p*-value < 0.001). On the other hand, no publication bias was reasonably associated with attack rates (Figure 8a; t = 0.65, df = 28, *p*-value = 0.519).

## 4. Discussion

### 4.1. Summary of the Main Findings

In this systematic review and meta-analysis, we conveyed the evidence from a total of 30 studies [38,43,52,60,62,74,75,76,77,78,79,80,81,82,83,84,85,86,87,88,89,90,91,92,93,94,95,96,97,98], including data from 20,067 BMT recipients, managed between 1989 and 2017, mostly from North America (USA and Canada) and the United Kingdom. A total of 821 cases of RSV infections were collected, for an attack rate of 5.40 per 100 people (95%CI 3.81 to 7.60) and an incidence rate of 14.77 per 1000 person-years (95%CI 9.43 to 20.11). The risk for RSV infection was higher among studies published between 2010 and 2014 compared to the more recent ones (RR 1.665, 95%CI 1.366 to 2.029), and in reports including only adults (RR 1.846, 95%CI 1.580 to 2.162) and only children (RR 1.264, 95%CI 1.054 to 1.517) compared to reports including both children and adults. A pooled CFR of 7.28% (95%CI 4.94 to 10.60) was calculated, with no substantial differences between studies only collecting data on children, on adults, and on both children and adults. Where data on both adults and children were provided, the former exhibited increased odds for developing RSV infection compared to adults (OR 2.941, 95%CI 1.689 to 5.122). Even though previous reports suggested a particularly dismal prognosis for RSV infections in recipients of allogenic BMT [18,44], no effective correlation was found between the proportion of allogenic BMT, attack rate (rho = 0.311, *p* = 0.139), incidence (rho = −0.042, *p* = 0.846), the proportion of LRTI cases (rho = −0.235, *p* = 0.280), and even CFR (rho = 0.085, *p* = 0.693). Also, the proportion of pediatric cases was not actually correlated with the main findings, but with the incidence rate (rho = −0.433, *p* = 0.044). On the other hand, the proportion of LRTI correlated well with CFR (rho = 0.445, *p* = 0.018).

### 4.2. Interpretation of Key Results

Since the early 1990s, RSV has been acknowledged as a common pathogen in immunocompromised patients, and is the most frequently identified viral respiratory tract infection agent in many observational studies on BMT recipients [105]. In our study, the occurrence of RSV infections was substantially higher than that of other respiratory pathogens, including influenza (5.40 per 100 patients, 95%CI 3.81 to 7.60 vs. 2.65 per 100 patients, 95%CI 1.53 to 4.54; RR 0.518; 95%CI 0.446 to 0.601), HAdV (2.10 per 100 patients, 95%CI 1.06 to 4.14; RR 0.679, 95%CI 0.553 to 0.830), and hMPV (1.77 per 100 patients, 95%CI 0.70 to 4.49; RR 0.536, 95%CI 0.438 to 0.655). In other words, RSV was ultimately characterized not only as a quite common respiratory pathogen, but also as more common than other viral agents, with a relatively high CFR. This specific finding was somewhat unexpected. Even though morbidity, mortality, and lethality estimates of RSV varied across the studies, RSV infections in healthy children and adults were usually characterized by low or even very low CFR; for instance, in a recent systematic review from Bylsma et al. [106], CFR ranged from 0.0% to 1.7% among US infants and children under 5 years of age, while CFR was estimated by Celante et al. [107] to be 6.6% among elderly patients hospitalized for RSV. On the other hand, previous reports on recipients of BMT suggested that RSV infections in these immunocompromised patients are characterized by an unusually high rate of progression from URTI to LRTI (i.e., 40 to 60%), with resulting mortality rates that in certain series have reached up to 60% [108,109] or even 80% [110,111,112,113]. Interestingly, the increased mortality associated with RSV could be due to both direct and indirect effects of the viral infection. On the one hand, LRTI due to RSV infections causes direct impairments to respiratory gas exchanges, with a resulting need for high-dose steroids at the time of LRTI infection, oxygen requirements, and mechanical ventilation [114]. On the other hand, tissue damage that is observed in the ciliated airway epithelium in response to RSV infections increases the risk for coinfections with other viruses and superinfection with bacteria or fungi, which in turn increase the risk for a very dismal prognosis [114].

Even though, in our estimate, no increased risk was seemingly associated with the proportion of allogenic BMT cases, earlier studies suggested that the occurrence of RSV infections was four to eight times higher in allogenic compared to autologous BMT (i.e., 3.5% to 8.8% vs. 0.4 to 1.5% attack rate) [105], and these results are highly consistent with more recent observational studies that have linked the high risk for RSV infection to chemotherapy and not only to the underlying disease [34,115]. Even in the FLUVAC trial, a large prospective observational study on influenza vaccine conducted in six French hospitals over three influenza seasons on adults with solid cancer and immunosuppressive treatment showed an increased risk of developing RSV infection (aOR 2.1, 95%CI 1.1 to 4.1) and 2.0 (1.1 to 3.8), respectively [116]. Not coincidentally, there is consolidated evidence that the share of RSV infections progressing from URTI to LRTI is particularly high during the pre-engraftment neutropenic period or ≤1 month post-transplant compared to during post-engraftment [86,108,117].

Another feature of RSV infections in BMT is that we are dealing with patients that are usually isolated from other patients but are also placed in close proximity to each other on dedicated wards [34,43]. In other words, while RSV is usually considered a community-acquired infection, commonly seen in the outpatient setting, especially during the respiratory viral season [110,118], the transmission of RSV in the healthcare settings is well documented [22]. In fact, a previous study suggested that nosocomial transmission may be responsible for approximately 50% of all cases [119], and even the large majority of cases included in the present review were reasonably nosocomial ones. With the notable exception of the study from Mikulska et al. [95], the main causes of the high incidence and attack rates should be identified in the characteristics of RSV infection in immunocompromised patients and in healthy healthcare staff. First, BMT recipients may have difficulties in clearing the virus because of their immunosuppressed state, and therefore have prolonged periods of viral shedding [94,120,121,122] whose occurrence is even more pronounced in individuals with prior allogenic transplantation and mismatched donor transplant (median duration of viral shedding for 80 days, range 35 to 334) [123]. Second, some transplant candidate pre-existing conditions have been characterized as risk factors for RSV, including smoking history, age > 65 years, conditioning with high-dose total body irradiation, myeloablative therapy, and the long duration of lymphopenia, while absolute lymphocyte count ≤ 100/mm^3^ at the time of upper respiratory infection onset has been associated with an increased risk for the progression of URTI to LRTI and pneumonia, with an absolute lymphocyte count > 1000/mm^3^ otherwise protective against progression [43,124]. Third, the clinical presentation of viral respiratory tract infection is often nonspecific [114], and only recently some specific CT scan features have been specifically analyzed [125]. Resulting diagnostic delays may, in turn, contribute to the high CFR [114]. Moreover, the healthcare staff is usually composed of adults with previous encounters with RSV, and who could develop self-limited and pauci-symptomatic infections [126,127,128], with 15% to 20% of healthcare providers possibly shedding RSV, and this figure can increase to 50% during community outbreaks [129,130]. In fact, in an earlier report from Taylor et al. [94] on the RSV season 1995–1996, eight out of ten cases of RSV occurring in a BMT unit in Bristol, UK, had identical RNA sequences, suggesting that the patients had become infected with the same strain of the pathogen, which therefore circulated widely.

### 4.3. Generalizability

**Figure A9:**
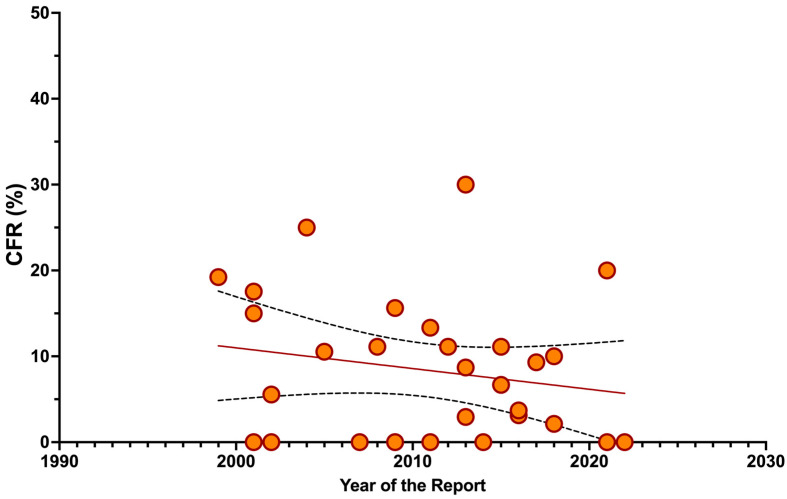
Time trend of the case fatality ratio (CFR). A non-significant negative association was eventually identified (rho = −0.178; *p* = 0.346) [46,51,61,68,70,80,81,82,83,84,85,86,87,88,89,90,91,92,93,94,95,96,97,98,99,100,101,102,103,104].

Our study included evidence from a quite extensive timeframe (1989 to 2021), mostly from North America [46,51,61,81,83,84,86,87,89,92,94,97,98,100,101,104], the UK, and Continental Europe [51,68,70,80,88,91,93,94,95,96,97], and more limited data from other settings [82,85,90,99,103]. Therefore, our pooled sample was quite heterogenous, not only in terms of potential exposure to the respiratory pathogens, but also for the changing landscape of bone marrow transplantation [131]. Since 1990, not only have more and more transplant centers been established worldwide, but major advances have been implemented in the recruitment of potential donors, as well as in the definition of conditioning regimes and preventive interventions aimed at reducing infection rates in BMT recipients [132]. Not coincidentally, the CFR for RSV-related infections was highest for studies published before 2005 (14.29%), decreasing in the following years to 7.46% (2005 to 2009), 7.19% (2010 to 2014), and eventually to 6.01 (2015 onwards), even though the very same timeframe exhibited a quite different trend in terms of attack rates, with the highest figures for 2010 to 2014 (7.18%), followed by 2015 onwards (4.31%) (Appendix A Figure A2 and Figure A9). The high risk for RSV infections between 2010 and 2014 was quite unexpected, as the global trend for RSV infections was not associated with an increased occurrence of the pathogen in the general population [4,5,8,127], while the pandemic 2009/H1N1 and claims about the reduced efficacy of the 2014–2015 influenza vaccine [85,87,102,103] encouraged the application of accurate preventive strategies. Consequently, the potential generalizability of pooled results should be preventively questioned. Similarly, it should be considered that, because of the poor outcome of SARS-CoV-2 infection in BMT recipients [133], preventive measures and standard operating procedures implemented by transplant centers have been extensively improved. As non-pharmaceutical interventions (i.e., actions, apart from getting vaccinated and taking medicine, that people and communities can take to help slow the spread of respiratory illnesses) [134,135,136] have been shown to be particularly effective in limiting the occurrence of all respiratory illnesses [137], the results collected before 2020 could only be somewhat representative of the ongoing risk for RSV infections in BMT centers. In fact, only the study of Samad et al. [99] included data from the pandemic settings, but the recruitment of new cases was interrupted by February 2020, making it limited as a representation of post-pandemic settings.

### 4.4. Limits and Implications for Future Studies

Even though our study provides a real-world estimate of RSV infections in a very high-risk subset of immunodeficient patients (i.e., recipients of BMT), being of potential significance for both Public Health and Healthcare professionals, our study is affected by several significant shortcomings that should be taken into account.

For one, even though most included studies were of appropriate or even of high quality, including four high-quality reports [83,90,92,93], some common and significant shortcomings must be addressed as they compromised both the accuracy and generalizability of the reported results, as otherwise stressed by the high heterogeneity we were able to identify across the whole of the pooled studies. On the one hand, a substantial share of studies did not accurately report the demographics of the whole sample [81,82,86,103], particularly when dealing with non-RSV cases [46,61,70,80,84,88,98,100,101,104]. On the other hand, when studies included both pediatric and adult cases, as well as autologous and allogenic transplantation, the reporting system did not regularly allow an accurate analysis of attack rates in these subgroups, impairing our analysis due to the lack of accurate calculation of corresponding pooled attack rates and CFR [80,81,89,91,97,100,101].

Second, it is unclear how many patients received palivizumab as either a preventive or therapeutic option. Palivizumab is a mAb which inhibits the activity of the F protein on the RSV envelope [138,139,140]. RSV immunoprophylaxis based on palivizumab has been approved for infants and young children from high-risk groups [127,128]. Even though the use of palivizumab in other settings, and particularly among older adults, has not been ascertained through specifically designed clinical trials [44], some suggest that it may represent a safe option for RSV prophylaxis among adult BMT [141], with some reports hinting at a potential use as a therapeutic option [142]. Overall, only 5 studies reported on the use of palivizumab [61,92,98,99,104], for a total of 30 cases over a total pool of 150 RSV cases (20.0%) and 3168 BMT recipients (0.94%). The limited use of palivizumab among BMT is reasonably based on two main shortcomings of this mAb. On the one hand, despite its proven efficacy [140,143] and its long stay on the international market, it remains an expensive medication, with a weight-dependent dose (i.e., 15 mg/kg) during the months characterized by a high circulation of the pathogen (“RSV season”) of up to five consecutive doses [127,140,143,144,145,146], and the cost for 100 mg vials ranging from around USD 900 to USD 1900 [4,5,24,25,26,28]; its systematic delivery in a 70 kg adult could therefore require charges ranging from around USD 9450 to USD 19,950 per month, which would scarcely be affordable even in high-income healthcare settings. More recently, the extended half-life of recombinant mAb nirsevimab (MEDI8897; commercial name: Beyfortus^®^; SANOFI Winthrop Industrie, Gentilly, France) has been approved by the Food and Drug Administration of the USA and European Medicine Agency (EMA) for the prevention of RSV-associated LRTI [23,57,147]. Similar to the palivizumab, current indications for nirsevimab are limited to newborns and infants from birth during their first RSV season [148,149], with a recommended dose of 50 mg for infants with body weight <5 kg and 100 mg for infants with body weight ≥ 5 kg, both delivered as a single dose [59,127,150,151], with no indications for adults and elderly patients. Recently, a single dose of 200 mg for infants aged 2 years or more still considered at high risk for RSV infections (children affected by: chronic lung disease of prematurity, hemodynamically significant congenital heart disease, immunodeficiency, etc.) has been taken into account [58,152]. Unfortunately, available studies on RSV prevention among for adults and elderly patients are mostly based on vaccines [25,153,154,155], but their reliability among BMT recipients is substantially impaired by the decline in antibody titers within weeks of the transplant and the limited response of the immune system to conventional immunization strategies during the high-risk pre-engraftment and early post-engraftment phases [156].

**Figure A10:**
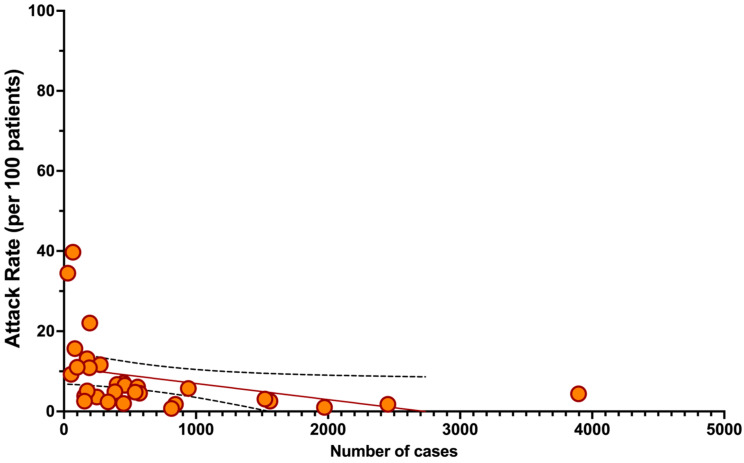
Correlation between attack rate for respiratory syncytial virus infections and number of cases included in the studies. A significant negative association was eventually identified (rho = −0.663; *p* < 0.001) [46,51,61,68,70,80,81,82,83,84,85,86,87,88,89,90,91,92,93,94,95,96,97,98,99,100,101,102,103,104].

**Figure A11:**
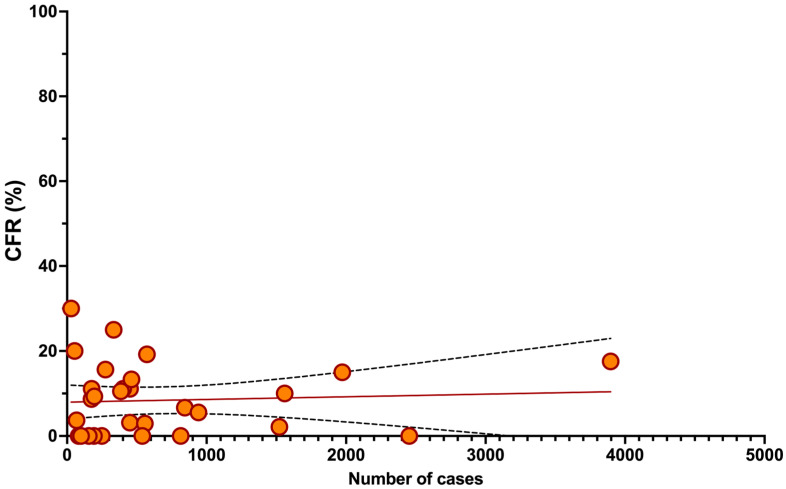
Correlation between case fatality ratio (CFR) for respiratory syncytial virus infections and number of cases included in the studies. A non-significant association was eventually identified (rho = 0.048; *p* = 0.803) [46,51,61,68,70,80,81,82,83,84,85,86,87,88,89,90,91,92,93,94,95,96,97,98,99,100,101,102,103,104].

Third, collected studies were not only quite heterogenous regarding timeframe and geographical settings, but also in sample size and reporting strategy. For instance, demographic data were not consistently provided by all retrieved studies, impairing the accurate appraisal of individual risk factors for developing RSV and other viral respiratory infections. Most notably, only five studies benefited from a prospective design, therefore being specifically tailored for collecting data on BMT recipients and respiratory infections [85,89,95,96,99], while only three studies provided detailed data on both children and adults [81,94,101]. As a consequence, corresponding ORs were calculated on a relatively small subset of cases, and corresponding estimates have to be cautiously assessed. In fact, nearly all reported studies were observational ones, and no preventive sample size calculations were systematically performed. Not coincidentally, some small study effects was suggested, particularly for attack rate, and a negative association was found between sample size and attack rate (i.e., the greater the size of the sample, the lower the eventual attack rate; Appendix A, Figure A10), while sample size was not correlated with the occurrence of LRTI over URTI (Appendix A, Figure A3) and CFR (Appendix A, Figure A11). As high attack rates, incidence rates, and CFR were identified in some reports, the reliability and generalizability of parent studies should therefore be carefully evaluated. In turn, our meta-analysis was affected by the very same shortcomings.

## 5. Conclusions

In conclusion, our systematic review and meta-analysis confirm earlier reports hinting at high attack rates, incidence rates, and CFR for RSV infections in BMT recipients. Even though the collected evidence was affected by some publication bias and high heterogeneity, our results collectively suggest that preventive interventions should be regularly put in place, enhancing the suspicion index for RSV infections even in highly controlled settings such as transplantation centers. Therefore, a proper preventive approach to BMT cases could encompass improved testing strategies with the periodic assessment of respiratory pathogens among newly admitted individuals, with new and effective preventive options such as mAb, although its delivery among adults and elderly patients still remains to be ascertained. Moreover, because of the noticeable CFR, specifically designed anti-viral drugs could substantially improve the prognosis of BMT recipients affected by RSV infections.

## Figures and Tables

**Table 1:** PECO worksheet [71,72].

Item	Definition
Population of interest	Individuals having received bone marrow transplant
Exposure	The occurrence of Respiratory Syncytial Virus (RSV) infection
Control/comparator	in children and adults Comparison to other respiratory pathogens (influenza, adenovirus, and human metapneumovirus)
Outcome	Occurrence of RSV infections, RSV-related lower respiratory tract infections and RSV-related deaths

**Table 2:** Summary of included studies.

Study	Country	Timeframe (Month–Year)	Design	BMT (N.)	Pediatric Cases (N., %)	Allogenic BMT (N., %)	RSV Cases					Flu (N.)	HAdV (N.)	hMPV (N.)
							Cases (N.)	Attack Rate (%)	LRTI (N., %)	Deaths (N.)	CFR (%)			
Avetysian et al., 2009 [80]	Sweden	01/2000–12/2007	S, R	275	NA	275, 100%	32	11.64%	14, 43.75%	5	15.63%	-	-	-
Campbell et al., 2015 [81]	USA	12/2005–02/2010	S, R	451	52, 11.53%	451, 100%	9	2.00%	0, -	1	11.11%	10	5	4
Chakrabarti et al., 2002 [51]	UK	06/1997–08/2001	S, R	83	0, -	83, 100%	13	15.66%	6, 46.15%	0	-	5	-	-
Choi et al., 2013 [82]	Korea	01/2007–03/2010	S, R	175	175, 100%	96, 54.86%	23	13.14%	12, 52.17%	2	8.70%	4	12	1
El-Bietar et al., 2016 [61]	USA	06/2008–12/2014	S, R	450	450, 100%	450, 100%	32	7.11%	6, 18.75%	1	3.13%	-	-	-
Fisher et al., 2018 [83]	USA	01/2010–06/2013	M, R	1560	1560, 100%	1144, 73.33%	40	2.56%	13, 32.50%	4	10.00%	29	-	17
Garrett Nichols et al., 2001 [84]	USA	1989–1999	S, R	3897	NA	NA	171	4.39%	68, 39.77%	30	17.54%	-	-	-
Gaytan Morales et al., 2021 [85]	Mexico	01/2017–12/2019	S, P	54	54, 100%	11, 20.37%	5	9.26%	5, 100%	1	20.00%	7	4	4
Ghosh et al., 2001 [86]	USA	11/1992–11/2000	S, R	249	0, -	249, 100%	9	3.61%	5, 55.56%	0	-	-	-	-
Gueller et al., 2013 [68]	Germany	10/2011–04/2012	S, R	29	0, -	29, 100%	10	34.48%	5, 50.00%	3	30.00%	-	-	-
Hutspardol et al., 2015 [87]	Canada	01/2000–12/2012	S, R	844	844, 100%	491, 58.18%	15	1.78%	8, 53.33%	1	6.67%	12	4	2
Khanna et al., 2008 [88]	Switzerland	02/2002–04/2007	S, R	402	NA	283, 70.40%	27	6.72%	7, 25.93%	3	11.11%	-	-	-
Kuypers et al., 2009 [89]	USA	12/2000–06/2004	S, P	157	NA	NA	6	3.82%	NA	0	0.00%	3	0	8
Lavergne et al., 2011 [46]	Canada	07/1999–06/2003	S, R	461	0, -	293, 63.56%	30	6.51%	16, 53.33%	4	13.33%	-	-	-
Lee et al., 2012 [90]	Korea	01/2007–08/2009	S, R	176	176, 100%	82, 46.59%	9	5.11%	NA	1	11.11%	-	1	-
Ljungman et al., 2001 [91]	Various	10/1997–10/1998	M, R	1973	NA	819, 41.51%	20	1.01%	14, 70.00%	3	15.00%	16	-	-
Lo et al., 2013 [92]	USA	01/1993–03/2006	S, R	557	557, 100%	NA	34	6.10%	14, 41.18%	1	2.94%	6	24	-
Martino et al., 2005 [93]	Spain	09/1999–10/2003	S, R	386	0, -	172, 44.56%	19	4.92%	11, 57.89%	2	10.53%	39	11	16
McCarthy et al., 1999 [94]	UK	09/1987–08/1998	S, R	572	381, 66.61%	474, 82.87%	26	4.55%	15, 57.69%	5	19.23%	-	-	-
McCoy et al., 2011 [104]	USA	09/2006–04/2009	S, R	539	0, -	196, 36.36%	26	4.82%	13, 50.00%	0	-	-	-	-
Mikulska et al., 2014 [95]	Italy	01/2011–03/2011	S, P	193	0, -	127, 65.80%	21	10.88%	2, 9.52%	0	-	20	3	-
Moret et al., 2021 [96]	Switzerland	11/2015–04/2016 11/2016–04/2017	S, P	156	0, -	0, -	4	2.56%	0, -	0	-	3	1	1
Peck et al., 2007 [97]	UK	12/2000–06/2004	S, R	814	NA	NA	6	0.74%	1, 16.67%	0	-	4	-	6
Rowan et al., 2018 [98]	USA	01/2010–12/2014	M, R	1522	1522, 100%	NA	47	3.09%	9, 19.15%	1	2.13%	-	-	-
Samad et al., 2022 [99]	India	01/2017–08/2021	S, P	100	0, -	NA	11	11.00%	2, 18.18%	0	-	-	-	-
Schiffer et al., 2009 [100]	USA	12/1997–03/2005	S, R	2453	NA	1620, 66.04%	44	1.79%	12, 27.27%	0	-	30	-	-
Schleuning et al., 2004 [70]	Germany	07/1998–06/2001	S, R	334	NA	334, 100%	8	2.40%	4, 50.00%	2	25.00%	-	16	-
Small et al., 2002 [101]	USA	01/1994–12/1999	S, R	942	154, 16.35%	548, 58.17%	54	5.73%	25, 46.30%	3	5.56%	-	-	-
Wang et al., 2017 [102]	Singapore	12/2010–10/2012	S, R	195	11, 5.64%	195, 100%	43	22.05%	12, 27.91%	4	9.30%	25	21	46
Yue et al., 2016 [103]	China	03/2011–02/2013	S, R	68	0	68, 100%	27	39.71%	16, 59.26%	1	3.70%	-	-	-

Note: S = single center; M = multi-center; P = prospective; R = retrospective; NA = not available/not provided; BMT = bone marrow transplantation; CFR = case fatality ratio; LRTI = lower respiratory tract infections; Flu = influenza virus infection; RSV = respiratory syncytial virus infection; HAdV = adenovirus infection; hMPV = human metapneumovirus infection.

**Table 3:** Summary of the collected studies on Respiratory Syncytial Virus (RSV) infections in recipients of bone marrow transplants (BMT).

**Collected Studies**	N.	30
**Collected cases of BMT**	N.	20,067
**Cases < 18 years of age**	N. (% of total cases)	5936 (29.58%)
**Cases ≥ 18 years of age**		3826 (19.07%)
**Undefined**		9762 (48.65%)
**Cases of allogenic transplantation**	N. (% of total cases)	8490 (42.31%)
**Cases of autologous transplantation**		4530 (22.57%)
**Undefined**		7047 (35.12%)
**Observation**	person-years	130,622.81
**Collected RSV cases**	N. (% of total cases)	821 (4.09%)
**LRTI cases**	N. (% of RSV cases)	351 (42.75%)
**RSV-related deaths**	N. (% of RSV cases)	78 (9.5%)
**Treatment with Palivizumab**	N. (% of RSV cases)	30 (3.65%)
**Cases sampled for Influenza**	N.	10,051
**Collected Influenza cases**	N. (% of sampled cases)	213 (2.12%)
**Cases sampled for Adenovirus**	N.	3678
**Collected Adenovirus cases**	N. (%of sampled cases)	102 (2.77%)
**Cases sampled for hMPV**	N.	4792
**Collected hMPV cases**	N. (%of sampled cases)	105 (2.19%)

Note: hMPV = human metapneumovirus; LRTI = lower respiratory tract infection.

**Table 4:** Characteristics of RSV cases by setting of the study.

	Total (No./20,067, %)	Positive (No./821, %)	Crude Attack Rate (%)	Risk Ratio	95% Confidence Interval
Country					
Canada	1305, 6.50%	45, 5.48%	3.45%	0.933	0.691; 1.261
China	68, 0.34%	27, 3.29%	39.71%	10.784	7.915; 14.596
Germany	363, 1.81%	18, 2.19%	4.96%	1.342	0.848; 2.124
India	100, 0.50%	11, 1.34%	11.00%	2.978	1.693; 5.236
Italy	193, 0.96%	21, 2.56%	10.88%	2.945	1.948; 4.453
South Korea	175, 0.87%	23, 2.80%	13.14%	3.558	2.406; 5.260
Mexico	54, 0.27%	5, 0.61%	9.26%	2.506	1.082; 5.804
Singapore	195, 0.97%	43, 5.24%	22.05%	5.969	4.520; 7.885
Spain	386, 1.92%	19, 2.31%	4.92%	1.332	0.852; 2.084
Sweden	275, 1.37%	32, 3.90%	11.64%	3.150	2.248; 4.414
Switzerland	558, 2.78%	31, 3.78%	5.56%	1.504	1.056; 2.141
United Kingdom	1469, 7.32%	45, 5.48%	3.06%	0.829	0.614; 1.120
United States	12,777, 63.67%	472, 57.49%	3.69%	1.000	REFERENCE
Various (Europe)	1973, 9.78483%	20, 2.44%	1.01%	0.274	0.176; 0.428
Timeframe					
Before 2005	8050, 40.12%	301, 36.66%	3.74%	0.867	0.733; 1.025
2005–2009	4487, 22.36%	134, 16.32%	2.99%	0.682	0.553; 0.841
2010–2014	2130, 10.61%	153, 18.64%	7.18%	1.665	1.366; 2.029
2015 onwards	5400, 26.91%	233, 28.38%	4.31%	1.000	REFERENCE
Patients					
Adults and Pediatric	8166, 40.69%	248, 30.21%	3.04%	1.000	REFERENCE
Adults only	6563, 32.71%	368, 44.82%	5.61%	1.846	1.580; 2.162
Pediatric only	5338, 26.60%	205, 24.97%	3.84%	1.264	1.054; 1.517
Study Design					
Multi-center	5055, 25.19%	107, 13.03%	2.12%	0.445	0.364; 0.544
Single center	15,012, 74.81%	714, 86.97%	4.76%	1.000	REFERENCE

**Table 5:** Characteristics of RSV-related deaths by setting of the study.

	Total (No./821, %)	Deaths (No./78, %)	Case Fatality Ratio (%)	Risk Ratio	95% Confidence Interval
Country					
Canada	45, 5.48%	5, 6.41%	11.11%	1.279	0.532; 3.073
China	27, 3.29%	1, 1.28%	3.70%	0.426	0.061; 2.983
Germany	18, 2.19%	5, 6.41%	27.78%	3.198	1.436; 7.119
India	11, 1.34%	0, -	0	0.523	0.034; 7.974
Italy	21, 2.56%	0, -	0	0.274	0.017; 4.306
Korea	23, 2.80%	3, 3.85%	9.38%	1.502	0.502; 4.885
Mexico	5, 0.61%	1, 1.28%	20.00%	2.302	0.389; 13.616
Singapore	43, 5.24%	4, 5.13%	9.30%	1.071	0.403; 2.848
Spain	19, 2.31%	2, 2.56%	10.53%	1.212	0.316; 4.642
Sweden	32, 3.90%	5, 6.41%	15.63%	1.799	0.763; 4.236
Switzerland	31, 3.78%	3, 3.85%	9.68%	1.114	0.366; 3.396
United Kingdom	45, 5.48%	5, 6.41%	11.11%	1.279	0.532; 3.073
United States	472, 57.49%	41, 52.56%	8.69%	1.000	REFERENCE
Various (Europe)	20, 2.44%	3, 3.85%	15.00%	1.727	0.584; 5.103
Timeframe					
Before 2005	301, 36.66%	43, 55.13%	14.29%	2.378	1.333; 4.239
2005–2009	134, 16.32%	10, 12.82%	7.46%	1.242	0.567; 2.718
2010–2014	153, 18.64%	11, 14.10%	7.19%	1.197	0.558; 2.566
2015 onwards	233, 28.38%	14, 17.95%	6.01%	1.000	REFERENCE
Patients					
Adults and Pediatric	248, 30.21%	23, 29.49%	9.27%	1.000	REFERENCE
Adults only	368, 44.82%	43, 55.13%	11.68%	1.260	0.780; 2.036
Pediatric only	205, 24.97%	12, 15.38%	5.85%	0.631	0.322; 1.237
Study Design					
Multicenter	107, 13.03%	8, 10.26%	7.48%	0.763	0.378; 1.540
Single center	714, 86.97%	70, 89.74%	9.40%	1.000	REFERENCE

**Table 6:** Correlation (Spearman’s rank test) between the proportion of allogenic bone marrow transplantation (BMT) over collected cases, and pediatric cases (i.e., subjects < 18 y.o. at the time of the BMT) with attack rates for respiratory syncytial virus (RSV) infections, incidence rate, proportion of lower respiratory tract infections (LRTI), and case fatality rate (CFR).

	RSV Attack Rate	RSV Incidence Rate	Proportion of LRTI	CFR
Proportion of allogenic BMT cases	rho = 0.311 (95%CI −0.118 to 0.643) *p* = 0.139	rho = −0.042 (95%CI −0.448 to 0.379) *p* = 0.846	rho = −0.235 (95%CI −0.599 to 0.208) *p* = 0.280	rho = 0.085 (95%CI −0.341 to 0.482) *p* = 0.693
Proportion of pediatric cases	rho = −0.283 (95%CI −0.638 to 0.170) *p* = 0.201	rho = −0.433 (95%CI −0.729 to −0.001) *p* = 0.044	rho = −0.008 (95%CI −0.449 to 0.437) *p* = 0.974	rho = 0.317 (95%CI −0.134 to 0.659) *p* = 0.150

**Table 7:** Pooled attack rate per 100 patients of RSV, RSV-related upper respiratory tract infections (URTI), RSV-related lower respiratory tract infections (LRTI), influenza, adenovirus, and human metapneumovirus (hMPV) infections.

	Pooled Attack Rate per 100 Patients (95%CI)		τ^2^	I^2^ (95%CI)	Q	*p*
RSV	5.40	3.81; 7.60	0.966	94.4% (93.0; 95.6)	519.16	<0.001
RSV (URTI)	3.09	1.84; 5.15	1.894	93.0% (91.0; 94.6)	385.45	<0.001
RSV (LRTI)	1.90	1.20; 2.99	1.369	89.7% (86.4; 92.3)	263.38	<0.001
Influenza	2.65	1.53; 4.54	1.082	94.3% (92.1; 95.9)	246.14	<0.001
AdV	2.10	1.06; 4.14	1.188	83.1% (71.9; 89.9)	65.17	<0.001
hMTP	1.77	0.70; 4.49	1.986	95.8% (93.9; 97.1)	215.75	<0.001

**Table 8:** Pooled incidence per 1000 patient-years of RSV, RSV-related upper respiratory tract infections (URTI), RSV-related lower respiratory tract infections (LRTI), influenza, adenovirus (HAdV), and human metapneumovirus (hMPV) infections.

	Pooled Incidence per 1000 Person-Years (95%CI)		τ^2^	I^2^ (95%CI)	Q	*p*
RSV	14.77	9.43; 20.12	0.001	91.6% (89.1; 93.5)	345.84	<0.001
RSV (URTI)	5.31	3.62; 6.99	0.001	88.4% (84.4; 91.4)	232.55	<0.001
RSV (LRTI)	3.99	2.40; 5.58	0.001	83.1% (76.5; 87.8)	159.27	<0.001
Influenza	10.45	4.04; 16.86	0.001	89.1% (83.7; 92.7)	128.42	<0.001
HAdV	9.64	2.95; 16.32	0.001	86.9% (78.9; 91.9)	83.98	<0.001
hMPV	15.56	0.00; 33.93	0.001	90.5% (84.6; 94.1)	94.57	<0.001

## Data Availability

Data are available on request from the corresponding author.

## Supplementary Materials

The following supporting information can be downloaded at: https://www.mdpi.com/article/10.3390/idr16020026/s1, Table S1: PRISMA checklist.

## Appendix A

**Table A1:** Detailed reporting on the Risk of Bias (ROB) estimates for observational studies [68,99]. Analyses were performed according to the National Toxicology Program (NTP)’s Office of Health Assessment and Translation (OHAT) handbook and respective risk of bias (ROB) tool. Note: D1: possibility of selection bias; D2: exposure assessment; D3: outcome assessment; D4: confounding factors; D5: reporting bias; D6: other bias; : definitively high; : probably high; : probably low; : definitively low.

Study	RISK OF BIAS					
	D1	D2	D3	D4	D5	D6
Avetysian et al., 2009 [80]						
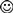
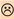
Campbell et al., 2015 [81]						
Chakrabarti et al., 2002 [51]						
Choi et al., 2013 [82]						
El-Bietar et al., 2016 [61]						
Fisher et al., 2018 [83]						
Garrett Nichols et al., 2001 [84]						
Gaytan Morales et al., 2021 [85]						
Ghosh et al., 2001 [86]						
Gueller et al., 2013 [68]						
Hutspardol et al., 2015 [87]						
Khanna et al., 2008 [88]						
Kuypers et al., 2009 [89]						
Lavergne et al., 2011 [46]						
Lee et al., 2012 [90]						
Ljungman et al., 2001 [91]						
Lo et al., 2013 [92]						
Martino et al., 2005 [93]						
McCarthy et al., 1999 [94]						
McCoy et al., 2011 [104]						
Mikulska et al., 2014 [95]						
Moret et al., 2021 [96]						
Peck et al., 2007 [97]						
Rowan et al., 2018 [98]						
Samad et al., 2022 [99]						
Schiffer et al., 2009 [100]						
Schleuning et al., 2004 [70]						
Small et al., 2002 [101]						
Wang et al., 2017 [102]						
Yue et al., 2016 [103]						

**Table A2:** Detailed description of the limits of the retrieved studies.

Study	Limits of the Study
Avetysian et al., 2009 [80]	report lacking data on non-RSV cases unclear reporting of demographic data
Campbell et al., 2015 [81]	did not report palivizumab delivery unclear reporting of demographic data by groups
Chakrabarti et al., 2002 [51]	unclear selection strategy (all participants from this single institution?)
Choi et al., 2013 [82]	did not report palivizumab delivery unclear reporting of demographic data by groups
El-Bietar et al., 2016 [61]	report lacking data on non-RSV cases unclear reporting of demographic data
Fisher et al., 2018 [83]	high-quality report
Garrett Nichols et al., 2001 [84]	report lacking data on non-RSV cases unclear reporting of demographic data unclear and confusing report about the eventual outcome
Gaytan Morales et al., 2021 [85]	unclear reporting of demographic data
Ghosh et al., 2001 [86]	only female patients unclear reporting of clinical and demographic data unclear and confusing report about the eventual outcome
Gueller et al., 2013 [68]	very small sample size (single outbreak) did not report palivizumab delivery unclear reporting of demographic data by groups
Hutspardol et al., 2015 [87]	high-quality report
Khanna et al., 2008 [88]	report lacking data on non-RSV cases other than influenza and para-influenza unclear reporting of demographic data
Kuypers et al., 2009 [89]	unclear reporting of clinical and demographic data
Lavergne et al., 2011 [46]	report lacking data on non-RSV cases unclear reporting of demographic data
Lee et al., 2012 [90]	did not report palivizumab delivery unclear reporting of demographic data by groups did not report the share of upper vs. lower respiratory tract
Ljungman et al., 2001 [91]	unclear reporting of clinical and demographic data unclear and confusing report about the eventual outcome
Lo et al., 2013 [92]	unclear reporting of demographic data by groups
Martino et al., 2005 [93]	high-quality report
McCarthy et al., 1999 [94]	high-quality report
McCoy et al., 2011 [104]	report lacking data on non-RSV cases unclear reporting of demographic data
Mikulska et al., 2014 [95]	outpatients doubtful reporting of main clinical features and demographic data
Moret et al., 2021 [96]	unclear reporting of demographic data
Peck et al., 2007 [97]	unclear reporting of clinical and demographic data
Rowan et al., 2018 [98]	report lacking data on non-RSV cases
Samad et al., 2022 [99]	unclear definition of the sample size unclear reporting of autologous vs. allogenic transplants
Schiffer et al., 2009 [100]	report lacking data on non-RSV cases other than influenza and para-influenza unclear reporting of demographic data
Schleuning et al., 2004 [70]	report lacking data on non-RSV cases other than influenza and para-influenza unclear reporting of demographic data
Small et al., 2002 [101]	report lacking data on non-RSV cases other than influenza and para-influenza unclear reporting of demographic data, particularly on autologous transplant
Wang et al., 2017 [102]	did not report palivizumab delivery
Yue et al., 2016 [103]	did not report palivizumab delivery unclear reporting of demographic data

## References

[1] Langedijk A.C., Harding E.R., Konya B., Vrancken B., Lebbink R.J., Evers A., Willemsen J., Lemey P., Bont L.J. (2021). A Systematic Review on Global RSV Genetic Data: Identification of Knowledge Gaps. Rev. Med. Virol..

[2] Nam H.H., Ison M.G. (2019). Respiratory Syncytial Virus Infection in Adults. BMJ.

[3] Troeger C., Blacker B., Khalil I.A., Rao P.C., Cao J., Zimsen S.R.M., Albertson S.B., Deshpande A., Farag T., Abebe Z. (2018). Estimates of the Global, Regional, and National Morbidity, Mortality, and Aetiologies of Lower Respiratory Infections in 195 Countries, 1990–2016: A Systematic Analysis for the Global Burden of Disease Study 2016. Lancet Infect. Dis..

[4] Shi T., McAllister D.A., O’Brien K.L., Simoes E.A.F., Madhi S.A., Gessner B.D., Polack F.P., Balsells E., Acacio S., Aguayo C. (2017). Global, Regional, and National Disease Burden Estimates of Acute Lower Respiratory Infections Due to Respiratory Syncytial Virus in Young Children in 2015: A Systematic Review and Modelling Study. Lancet.

[5] Nair H., Theodoratou E., Rudan I., Nokes D.J., Ngama HND M., Munywoki P.K., Dherani M., Nair H., James Nokes D., Gessner B.D. (2010). Global Burden of Acute Lower Respiratory Infections Due to Respiratory Syncytial Virus in Young Children: A Systematic Review and Meta-Analysis. Lancet.

[6] Luo W., Liu Q., Zhou Y., Ran Y., Liu Z., Hou W., Pei S., Lai S. (2023). Spatiotemporal Variations of “Triple-Demic” Outbreaks of Respiratory Infections in the United States in the Post-COVID-19 Era. BMC Public Health.

[7] Patel T.A., Jain B., Raifman J. (2023). Revamping Public Health Systems: Lessons Learned From the Tripledemic. Am. J. Prev. Med..

[8] Shi T., Denouel A., Tietjen A.K., Campbell I., Moran E., Li X., Campbell H., Demont C., Nyawanda B.O., Chu H.Y. (2021). Global Disease Burden Estimates of Respiratory Syncytial Virus-Associated Acute Respiratory Infection in Older Adults in 2015: A Systematic Review and Meta-Analysis. J. Infect. Dis..

[9] Regassa B.T., Gebrewold L.A., Mekuria W.T., Kassa N.A. (2023). Molecular Epidemiology of Respiratory Syncytial Virus in Children with Acute Respiratory Illnesses in Africa: A Systematic Review and Meta-Analysis. J. Glob. Health.

[10] Youssef Y., Chmaisse A., Boutros C., Chamseddine S., Fayad D., Zaraket H., Dbaibo G. (2021). The Burden of Respiratory Syncytial Virus (RSV) Infection in the Middle East and North Africa (MENA) Region across Age Groups: A Systematic Review. Vaccine.

[11] Li Y., Wang X., Blau D.M., Caballero M.T., Feikin D.R., Gill C.J., Madhi S.A., Omer S.B., Simões E.A.F., Campbell H. (2022). Global, Regional, and National Disease Burden Estimates of Acute Lower Respiratory Infections Due to Respiratory Syncytial Virus in Children Younger than 5 Years in 2019: A Systematic Analysis. Lancet.

[12] Du Y., Yan R., Wu X., Zhang X., Chen C., Jiang D., Yang M., Cao K., Chen M., You Y. (2023). Global Burden and Trends of Respiratory Syncytial Virus Infection across Different Age Groups from 1990 to 2019: A Systematic Analysis of the Global Burden of Disease 2019 Study. Int. J. Infect. Dis..

[13] Nguyen-Van-tam J.S., O’leary M., Martin E.T., Heijnen E., Callendret B., Fleischhackl R., Comeaux C., Tran T.M.P., Weber K. (2022). Burden of Respiratory Syncytial Virus Infection in Older and High-Risk Adults: A Systematic Review and Meta-Analysis of the Evidence from Developed Countries. Eur. Respir. Rev..

[14] Weigl J.A.I., Puppe W., Schmitt H.J. (2001). Incidence of Respiratory Syncytial Virus-Positive Hospitalizations in Germany. Eur. J. Clin. Microbiol. Infect. Dis..

[15] Esposito S., Scarselli E., Lelii M., Scala A., Vitelli A., Capone S., Fornili M., Biganzoli E., Orenti A., Nicosia A. (2016). Antibody Response to Respiratory Syncytial Virus Infection in Children <18 Months Old. Hum. Vaccin. Immunother..

[16] Tabatabai J., Ihling C.M., Rehbein R.M., Schnee S.V., Hoos J., Pfeil J., Grulich-Henn J., Schnitzler P. (2022). Molecular Epidemiology of Respiratory Syncytial Virus in Hospitalised Children in Heidelberg, Southern Germany, 2014–2017. Infect. Genet. Evol..

[17] Hall C.B., Weinberg G.A., Blumkin A.K., Edwards K.M., Staat M.A., Schultz A.F., Poehling K.A., Szilagyi P.G., Griffin M.R., Williams J.V. (2013). Respiratory Syncytial Virus-Associated Hospitalizations among Children Less Than 24 Months of Age. Pediatrics.

[18] Abbas S., Raybould J.E., Sastry S., de la Cruz O. (2017). Respiratory Viruses in Transplant Recipients: More than Just a Cold. Clinical Syndromes and Infection Prevention Principles. Int. J. Infect. Dis..

[19] Bozzola E., Ciarlitto C., Guolo S., Brusco C., Cerone G., Antilici L., Schettini L., Piscitelli A.L., Chiara Vittucci A., Cutrera R. (2021). Respiratory Syncytial Virus Bronchiolitis in Infancy: The Acute Hospitalization Cost. Front. Pediatr..

[20] Rha B., Curns A.T., Lively J.Y., Campbell A.P., Englund J.A., Boom J.A., Azimi P.H., Weinberg G.A., Staat M.A., Selvarangan R. (2020). Respiratory Syncytial Virus-Associated Hospitalizations among Young Children: 2015–2016. Pediatrics.

[21] Leader S., Kohlhase K. (2002). Respiratory Syncytial Virus-Coded Pediatric Hospitalizations, 1997 to 1999. Pediatr. Infect. Dis. J..

[22] Leader S., Kohlhase K., Pearlman M.H., Williams J.V., Engle W.A. (2003). Recent Trends in Severe Respiratory Syncytial Virus (RSV) among US Infants, 1997 to 2000. J. Pediatr..

[23] Hammitt L.L., Dagan R., Yuan Y., Baca Cots M., Bosheva M., Madhi S.A., Muller W.J., Zar H.J., Brooks D., Grenham A. (2022). Nirsevimab for Prevention of RSV in Healthy Late-Preterm and Term Infants. N. Engl. J. Med..

[24] Aliprantis A.O., Shaw C.A., Griffin P., Farinola N., Railkar R.A., Cao X., Liu W., Sachs J.R., Swenson C.J., Lee H. (2021). A Phase 1, Randomized, Placebo-Controlled Study to Evaluate the Safety and Immunogenicity of an MRNA-Based RSV Prefusion F Protein Vaccine in Healthy Younger and Older Adults. Hum. Vaccin. Immunother..

[25] Walsh E.E., Pérez Marc G., Zareba A.M., Falsey A.R., Jiang Q., Patton M., Polack F.P., Llapur C., Doreski P.A., Ilangovan K. (2023). Efficacy and Safety of a Bivalent RSV Prefusion F Vaccine in Older Adults. N. Engl. J. Med..

[26] Chatzis O., Darbre S., Pasquier J., Meylan P., Manuel O., Aubert J.D., Beck-Popovic M., Masouridi-Levrat S., Ansari M., Kaiser L. (2018). Burden of Severe RSV Disease among Immunocompromised Children and Adults: A 10 Year Retrospective Study. BMC Infect. Dis..

[27] Boattini M., Almeida A., Christaki E., Marques T.M., Tosatto V., Bianco G., Iannaccone M., Tsiolakkis G., Karagiannis C., Maikanti P. (2021). Severity of RSV Infection in Southern European Elderly Patients during Two Consecutive Winter Seasons (2017–2018). J. Med. Virol..

[28] Falsey A.R., Koval C., DeVincenzo J.P., Walsh E.E. (2017). Compassionate Use Experience with High-Titer Respiratory Syncytical Virus (RSV) Immunoglobulin in RSV-Infected Immunocompromised Persons. Transpl. Infect. Dis..

[29] Falsey A.R., Hennessey P.A., Formica M.A., Cox C., Walsh E.E. (2005). Respiratory Syncytial Virus Infection in Elderly and High-Risk Adults. N. Engl. J. Med..

[30] Ali A., Lopardo G., Scarpellini B., Stein R.T., Ribeiro D. (2020). Systematic Review on Respiratory Syncytial Virus Epidemiology in Adults and the Elderly in Latin America. Int. J. Infect. Dis..

[31] Nowalk M.P., D’Agostino H., Dauer K., Stiegler M., Zimmerman R.K., Balasubramani G.K. (2022). Estimating the Burden of Adult Hospitalized RSV Infection Including Special Populations. Vaccine.

[32] Narejos Pérez S., Ramón Torrell J.M., Põder A., Leroux-Roels I., Pérez-Breva L., Steenackers K., Vandermeulen C., Meisalu S., McNally D., Bowen J.S. (2023). Respiratory Syncytial Virus Disease Burden in Community-Dwelling and Long-Term Care Facility Older Adults in Europe and the United States: A Prospective Study. Open Forum Infect. Dis..

[33] Savic M., Penders Y., Shi T., Branche A., Pirçon J.Y. (2022). Respiratory Syncytial Virus Disease Burden in Adults Aged 60 Years and Older in High-Income Countries: A Systematic Literature Review and Meta-Analysis. Influenza Other Respir. Viruses.

[34] Chemaly R.F., Shah D.P., Boeckh M.J. (2014). Management of Respiratory Viral Infections in Hematopoietic Cell Transplant Recipients and Patients with Hematologic Malignancies. Clin. Infect. Dis..

[35] El Saleeby C.M., Somes G.W., DeVincenzo J.P., Gaur A.H. (2008). Risk Factors for Severe Respiratory Syncytial Virus Disease in Children with Cancer: The Importance of Lymphopenia and Young Age. Pediatrics.

[36] Torres J.P., Tapia L.I., Catalán P., De la Maza V., Mejías A. (2017). Intravenous Palivizumab in Respiratory Syncytial Virus Infection after Hematopoietic Stem Cell Transplant in Children. Pediatr. Blood Cancer.

[37] Permpalung N., Mahoney M.V., McCoy C., Atsawarungruangkit A., Gold H.S., Levine J.D., Wong M.T., LaSalvia M.T., Alonso C.D. (2019). Clinical Characteristics and Treatment Outcomes among Respiratory Syncytial Virus (RSV)-Infected Hematologic Malignancy and Hematopoietic Stem Cell Transplant Recipients Receiving Palivizumab. Leuk. Lymphoma.

[38] Madhi S.A., Cutland C.L., Downs S., Jones S., Van Niekerk N., Simoes E.A.F., Nunes M.C. (2018). Burden of Respiratory Syncytial Virus Infection in South African Human Immunodeficiency Virus (HIV)-Infected and HIV-Uninfected Pregnant and Postpartum Women: A Longitudinal Cohort Study. Clin. Infect. Dis..

[39] Zhang Y., Yuan L., Zhang Y., Zhang X., Zheng M., Kyaw M.H. (2015). Burden of Respiratory Syncytial Virus Infections in China: Systematic Review and Meta-Analysis. J. Glob. Health.

[40] Palmer L., Hall C.B., Katkin J.P., Shi N., Masaquel A.S., McLaurin K.K., Mahadevia P.J. (2010). Healthcare Costs within a Year of Respiratory Syncytial Virus among Medicaid Infants. Pediatr. Pulmonol..

[41] McLaurin K.K., Farr A.M., Wade S.W., Diakun D.R., Stewart D.L. (2016). Respiratory Syncytial Virus Hospitalization Outcomes and Costs of Full-Term and Preterm Infants. J. Perinatol..

[42] Mao Z., Li X., Dacosta-Urbieta A., Billard M.N., Wildenbeest J., Korsten K., Martinón-Torres F., Heikkinen T., Cunningham S., Snape M.D. (2023). Economic Burden and Health-Related Quality-of-Life among Infants with Respiratory Syncytial Virus Infection: A Multi-Country Prospective Cohort Study in Europe. Vaccine.

[43] Neemann K., Freifeld A. (2015). Respiratory Syncytial Virus in Hematopoietic Stem Cell Transplantation and Solid-Organ Transplantation. Curr. Infect. Dis. Rep..

[44] Villanueva D.D.H., Arcega V., Rao M. (2022). Review of Respiratory Syncytial Virus Infection among Older Adults and Transplant Recipients. Ther. Adv. Infect. Dis..

[45] Manothummetha K., Mongkolkaew T., Tovichayathamrong P., Boonyawairote R., Meejun T., Srisurapanont K., Phongkhun K., Sanguankeo A., Torvorapanit P., Moonla C. (2023). Ribavirin Treatment for Respiratory Syncytial Virus Infection in Patients with Haematologic Malignancy and Haematopoietic Stem Cell Transplant Recipients: A Systematic Review and Meta-Analysis. Clin. Microbiol. Infect..

[46] Lavergne V., Ghannoum M., Weiss K., Roy J., Béliveau C. (2011). Successful Prevention of Respiratory Syncytial Virus Nosocomial Transmission Following an Enhanced Seasonal Infection Control Program. Bone Marrow Transplant..

[47] Anderson N.W., Binnicker M.J., Harris D.M., Chirila R.M., Brumble L., Mandrekar J., Hata D.J. (2016). Morbidity and Mortality among Patients with Respiratory Syncytial Virus Infection: A 2-Year Retrospective Review. Diagn. Microbiol. Infect. Dis..

[48] Vakil E., Sheshadri A., Faiz S.A., Shah D.P., Zhu Y., Li L., Kmeid J., Azzi J., Balagani A., Bashoura L. (2018). Risk Factors for Mortality after Respiratory Syncytial Virus Lower Respiratory Tract Infection in Adults with Hematologic Malignancies. Transpl. Infect. Dis..

[49] Sheshadri A., Chemaly R.F., Alousi A.M., Shah P.K., Rondon G., Bashoura L., Kmeid J., Azzi J., Blanco D.W., Kaous M. (2019). Pulmonary Impairment after Respiratory Viral Infections Is Associated with High Mortality in Allogeneic Hematopoietic Cell Transplant Recipients. Biol. Blood Marrow Transplant..

[50] Renaud C., Xie H., Seo S., Kuypers J., Cent A., Corey L., Leisenring W., Boeckh M., Englund J.A. (2013). Mortality Rates of Human Metapneumovirus and Respiratory Syncytial Virus Lower Respiratory Tract Infections in Hematopoietic Cell Transplantation Recipients. Biol. Blood Marrow Transplant..

[51] Chakrabarti S., Avivi I., Mackinnon S., Ward K., Kottaridis P.D., Osman H., Waldmann H., Hale G., Fegan C.D., Yong K. (2002). Respiratory Virus Infections in Transplant Recipients after Reduced-Intensity Conditioning with Campath-1H: High Incidence but Low Mortality. Br. J. Haematol..

[52] Melgar M., Britton A., Roper L.E., Talbot K.H., Long S.S., Kotton C.N., Havers F.P. (2023). Use of Respiratory Syncytial Virus Vaccines in Older Adults: Recommendations of the Advisory Committee on Immunization Practices—United States, 2023. Morb. Mortal. Wkly. Rep..

[53] Fleming-Dutra K.E., Jones J.M., Roper L.E., Prill M.M., Ortega-Sanchez I.R., Moulia D.L., Wallace M., Godfrey M., Broder K.R., Tepper N.K. (2023). Use of the Pfizer Respiratory Syncytial Virus Vaccine During Pregnancy for the Prevention of Respiratory Syncytial Virus-Associated Lower Respiratory Tract Disease in Infants: Recommendations of the Advisory Committee on Immunization Practices-United States, 2023. Morb. Mortal. Wkly. Rep..

[54] Kampmann B., Madhi S.A., Munjal I., Simões E.A.F., Pahud B.A., Llapur C., Baker J., Pérez Marc G., Radley D., Shittu E. (2023). Bivalent Prefusion F Vaccine in Pregnancy to Prevent RSV Illness in Infants. N. Engl. J. Med..

[55] Papi A., Ison M.G., Langley J.M., Lee D.-G., Leroux-Roels I., Martinon-Torres F., Schwarz T.F., van Zyl-Smit R.N., Campora L., Dezutter N. (2023). Respiratory Syncytial Virus Prefusion F Protein Vaccine in Older Adults. N. Engl. J. Med..

[56] Andabaka T., Nickerson J.W., Rojas-Reyes M.X., Rueda J.D., Bacic Vrca V., Barsic B. (2013). Monoclonal Antibody for Reducing the Risk of Respiratory Syncytial Virus Infection in Children. Cochrane Database Syst. Rev..

[57] Griffin M.P., Yuan Y., Takas T., Domachowske J.B., Madhi S.A., Manzoni P., Simões E.A.F., Esser M.T., Khan A.A., Dubovsky F. (2020). Single-Dose Nirsevimab for Prevention of RSV in Preterm Infants. N. Engl. J. Med..

[58] Domachowske J.B., Chang Y., Atanasova V., Cabañas F., Furuno K., Nguyen K.A., Banu I., Kubiak R.J., Leach A., Mankad V.S. (2023). Safety of Re-Dosing Nirsevimab Prior to RSV Season 2 in Children with Heart or Lung Disease. J. Pediatr. Infect. Dis. Soc..

[59] Domachowske J., Madhi S.A., Simões E.A.F., Atanasova V., Cabañas F., Furuno K., Garcia-Garcia M.L., Grantina I., Nguyen K.A., Brooks D. (2022). Safety of Nirsevimab for RSV in Infants with Heart or Lung Disease or Prematurity. N. Engl. J. Med..

[60] Brady M.T., Byington C.L., Dele Davies H., Edwards K.M., Jackson M.A., Maldonado Y.A., Murray D.L., Orenstein W.A., Rathore M.H., Committee on Infectious Diseases and Bronchiolitis Guidelines Committee (2014). Updated Guidance for Palivizumab Prophylaxis among Infants and Young Children at Increased Risk of Hospitalization for Respiratory Syncytial Virus Infection. Pediatrics.

[61] El-Bietar J., Nelson A., Wallace G., Dandoy C., Jodele S., Myers K.C., Teusink A., Lane A., Davies S.M., Danziger-Isakov L. (2016). RSV Infection without Ribavirin Treatment in Pediatric Hematopoietic Stem Cell Transplantation. Bone Marrow Transplant..

[62] Molinos-Quintana A., Pérez-De Soto C., Gómez-Rosa M., Pérez-Simón J.A., Pérez-Hurtado J.M. (2013). Intravenous Ribavirin for Respiratory Syncytial Viral Infections in Pediatric Hematopoietic SCT Recipients. Bone Marrow Transplant..

[63] Marcelin J.R., Wilson J.W., Razonable R.R. (2014). Oral Ribavirin Therapy for Respiratory Syncytial Virus Infections in Moderately to Severely Immunocompromised Patients. Transpl. Infect. Dis..

[64] Chávez-Bueno S., Mejías A., Merryman R.A., Ahmad N., Jafri H.S., Ramilo O. (2007). Intravenous Palivizumab and Ribavirin Combination for Respiratory Syncytial Virus Disease in High-Risk Pediatric Patients. Pediatr. Infect. Dis. J..

[65] Gorcea C.M., Tholouli E., Turner A., Saif M., Davies E., Battersby E., Dignan F.L. (2017). Effective Use of Oral Ribavirin for Respiratory Syncytial Viral Infections in Allogeneic Haematopoietic Stem Cell Transplant Recipients. J. Hosp. Infect..

[66] Foolad F., Aitken S.L., Shigle T.L., Prayag A., Ghantoji S., Ariza-Heredia E., Chemaly R.F. (2019). Oral versus Aerosolized Ribavirin for the Treatment of Respiratory Syncytial Virus Infections in Hematopoietic Cell Transplant Recipients. Clin. Infect. Dis..

[67] Stamouli M., Tsonis I., Gkirkas K., Economopoulou C., Siafakas N., Pournaras S., Antoniadou A., Chondropoulos S., Karagiannidi A., Meletiadis J. (2021). Oral Ribavirin Is a Highly Effective Treatment for Lower Respiratory Tract Infections Due to Respiratory Syncytial Virus or Parainfluenza after Allogeneic Stem Cell Transplantation. Bone Marrow Transplant..

[68] Gueller S., Duenzinger U., Wolf T., Ajib S., Mousset S., Berger A., Martin H., Serve H., Bug G. (2013). Successful Systemic High-Dose Ribavirin Treatment of Respiratory Syncytial Virus-Induced Infections Occurring Pre-Engraftment in Allogeneic Hematopoietic Stem Cell Transplant Recipients. Transpl. Infect. Dis..

[69] Akhmedov M., Wais V., Sala E., Neagoie A., Nguyen T.M., Gantner A., von Harsdorf S., Kuchenbauer F., Schubert A., Michel D. (2020). Respiratory Syncytial Virus and Human Metapneumovirus after Allogeneic Hematopoietic Stem Cell Transplantation: Impact of the Immunodeficiency Scoring Index, Viral Load, and Ribavirin Treatment on the Outcomes. Transpl. Infect. Dis..

[70] Schleuning M., Buxbaum-Conradi H., Jäger G., Kolb H.J. (2004). Intravenous Ribavirin for Eradication of Respiratory Syncytial Virus (RSV) and Adenovirus Isolates from the Respiratory and/or Gastrointestinal Tract in Recipients of Allogeneic Hematopoietic Stem Cell Transplants. Hematol. J..

[71] Page M.J., McKenzie J.E., Bossuyt P.M., Boutron I., Hoffmann T.C., Mulrow C.D., Shamseer L., Tetzlaff J.M., Akl E.A., Brennan S.E. (2021). The PRISMA 2020 statement: An updated guideline for reporting systematic reviews. BMJ.

[72] Moher D., Liberati A., Tetzlaff J., Altman D.G., Altman D., Antes G., Atkins D., Barbour V., Barrowman N., Berlin J.A. (2009). Preferred Reporting Items for Systematic Reviews and Meta-Analyses: The PRISMA Statement. PLoS Med..

[73] NTP (2015). OHAT Risk of Bias Rating Tool for Human and Animal Studies.

[74] National Toxicology Program (2019). Handbook for Conducting a Literature-Based Health Assessment Using OHAT Approach for Systematic Review and Evidence Integration.

[75] Eick S.M., Goin D.E., Chartres N., Lam J., Woodruff T.J. (2020). Assessing Risk of Bias in Human Environmental Epidemiology Studies Using Three Tools: Different Conclusions from Different Tools. Syst. Rev..

[76] Von Hippel P.T. (2015). The Heterogeneity Statistic I2 Can Be Biased in Small Meta-Analyses. BMC Med. Res. Methodol..

[77] Imrey P.B. (2020). Limitations of Meta-Analyses of Studies with High Heterogeneity. JAMA Netw. Open.

[78] R Development Core Team (2010). R a Language and Environment for Statistical Computing: Reference Index.

[79] Haddaway N.R., Page M.J., Pritchard C.C., McGuinness L.A. (2022). PRISMA2020: An R Package and Shiny App for Producing PRISMA 2020-Compliant Flow Diagrams, with Interactivity for Optimised Digital Transparency and Open Synthesis. Campbell Syst. Rev..

[80] Avetisyan G., Mattsson J., Sparrelid E., Ljungman P. (2009). Respiratory Syncytial Virus Infection in Recipients of Allogeneic Stem-Cell Transplantation: A Retrospective Study of the Incidence, Clinical Features, and Outcome. Transplantation.

[81] Campbell A.P., Guthrie K.A., Englund J.A., Farney R.M., Minerich E.L., Kuypers J., Corey L., Boeckh M. (2015). Clinical Outcomes Associated with Respiratory Virus Detection before Allogeneic Hematopoietic Stem Cell Transplant. Clin. Infect. Dis..

[82] Choi J.H., Choi E.H., Kang H.J., Park K.D., Park S.S., Shin H.Y., Lee H.J., Ahn H.S. (2013). Respiratory Viral Infections after Hematopoietic Stem Cell Transplantation in Children. J. Korean Med. Sci..

[83] Fisher B.T., Danziger-Isakov L., Sweet L.R., Munoz F.M., Maron G., Tuomanen E., Murray A., Englund J.A., Dulek D., Halasa N. (2018). A Multicenter Consortium to Define the Epidemiology and Outcomes of Inpatient Respiratory Viral Infections in Pediatric Hematopoietic Stem Cell Transplant Recipients. J. Pediatr. Infect. Dis. Soc..

[84] Garrett Nichols W., Gooley T., Boeckh M. (2001). Community-Acquired Respiratory Syncytial Virus and Parainfluenza Virus Infections after Hematopoietic Stem Cell Transplantation: The Fred Hutchinson Cancer Research Center Experience. Biol. Blood Marrow Transplanat..

[85] Gaytán-Morales J.F., Castorena-Villa I., Cortés-Flores D.C., Avilés-Robles M.J., Sánchez-Huerta J.L., Ortiz-Navarrete V., Olvera-Gómez I., López-Martínez B., Parra-Ortega I. (2021). Respiratory Viral Infections in Pediatric Patients with Hematopoietic Stem Cell Transplantation. Bol. Med. Hosp. Infant. Mex..

[86] Ghosh S., Champlin R.E., Ueno N.T., Anderlini P., Rolston K., Raad I., Kontoyiannis D., Jacobson K., Luna M., Tarrand J. (2001). Infections Post Transplant Respiratory Syncytial Virus Infections in Autologous Blood and Marrow Transplant Recipients with Breast Cancer: Combination Therapy with Aerosolized Ribavirin and Parenteral Immunoglobulins. Bone Marrow Transplant..

[87] Hutspardol S., Essa M., Richardson S., Schechter T., Ali M., Krueger J., Fujii H., Egeler R.M., Gassas A. (2015). Significant Transplantation-Related Mortality from Respiratory Virus Infections within the First One Hundred Days in Children after Hematopoietic Stem Cell Transplantation. Biol. Blood Marrow Transplant..

[88] Khanna N., Widmer A.F., Decker M., Steffen I., Halter J., Heim D., Weisser M., Gratwohl A., Fluckiger U., Hirsch H.H. (2008). Respiratory Syncytial Virus Infection in Patients with Hematological Diseases: Single-Center Study and Review of the Literature. Clin. Infect. Dis..

[89] Kuypers J., Campbell A.P., Cent A., Corey L., Boeckh M. (2009). Comparison of Conventional and Molecular Detection of Respiratory Viruses in Hematopoietic Cell Transplant Recipients. Transpl. Infect. Dis..

[90] Lee J.H., Jang J.H., Lee S.H., Kim Y.J., Yoo K.H., Sung K.W., Lee N.Y., Ki C.S., Koo H.H. (2012). Respiratory Viral Infections during the First 28 Days after Transplantation in Pediatric Hematopoietic Stem Cell Transplant Recipients. Clin. Transplant..

[91] Ljungman P., Ward K.N., Crooks B., Parker A., Martino R., Shaw P.J., Brinch L., Brune M., De R., Camara L. (2001). Viral Infections Respiratory Virus Infections after Stem Cell Transplantation: A Prospective Study from the Infectious Diseases Working Party of the European Group for Blood and Marrow Transplantation. Bone Marrow Transplant..

[92] Lo M.S., Lee G.M., Gunawardane N., Burchett S.K., Lachenauer C.S., Lehmann L.E. (2013). The Impact of RSV, Adenovirus, Influenza, and Parainfluenza Infection in Pediatric Patients Receiving Stem Cell Transplant, Solid Organ Transplant, or Cancer Chemotherapy. Pediatr. Transplant..

[93] Martino R., Porras R.P., Rabella N., Williams J.V., Rámila E., Margall N., Labeaga R., Crowe J.E., Coll P., Sierra J. (2005). Prospective Study of the Incidence, Clinical Features, and Outcome of Symptomatic Upper and Lower Respiratory Tract Infections by Respiratory Viruses Is Adult Recipients of Hematopoietic Stem Cell Transplants for Hematologic Malignancies. Biol. Blood Marrow Transplant..

[94] Mccarthy A.J., Kingman H.M., Kelly C., Taylor G.S., Caul E.O., Grier D., Moppett J., Foot A., Cornish J.M., Oakhill A. (1999). The Outcome of 26 Patients with Respiratory Syncytial Virus Infection Following Allogeneic Stem Cell Transplantation. Bone Marrow Transplant..

[95] Mikulska M., Del Bono V., Gandolfo N., Dini S., Dominietto A., Di Grazia C., Bregante S., Varaldo R., Orsi A., Ansaldi F. (2014). Epidemiology of Viral Respiratory Tract Infections in an Outpatient Haematology Facility. Ann. Hematol..

[96] Moret F., Marschall J., Atkinson A., Farag S., Zimmerli S., Pabst T., Sommerstein R. (2021). Characteristics of Respiratory Virus Infections in Autologous Hematopoietic Stem Cell Transplantation Patients, a Prospective Study, Bern, Switzerland, 2015–2017. Infect. Dis..

[97] Peck A.J., Englund J.A., Kuypers J., Guthrie K.A., Corey L., Morrow R., Hackman R.C., Cent A., Boeckh M. (2007). Respiratory Virus Infection among Hematopoietic Cell Transplant Recipients: Evidence for Asymptomatic Parainfluenza Virus Infection. Blood.

[98] Rowan C.M., Gertz S.J., Zinter M.S., Moffet J., Bajwa R.P.S., Barnum J.L., Kong M. (2018). A Multicenter Investigation of Respiratory Syncytial Viral Infection in Children with Hematopoietic Cell Transplantation. Transpl. Infect. Dis..

[99] Samad S.A., Jethani J., Kumar L., Choudhary A., Brijwal M., Dar L. (2022). Respiratory Syncytial Virus Infection among Adults after Hematopoietic Stem Cell Transplantation. J. Glob. Infect. Dis..

[100] Schiffer J.T., Kirby K., Sandmaier B., Storb R., Corey L., Boeckh M. (2009). Timing and Severity of Community Acquired Respiratory Virus Infections after Myeloablative versus Non-Myeloablative Hematopoietic Stem Cell Transplantation. Haematologica.

[101] Small T.N., Casson A., Malak S.F., Boulad F., Kiehn T.E., Stiles J., Ushay H.M., Sepkowitz K.A. (2002). Viral Infections Respiratory Syncytial Virus Infection Following Hematopoietic Stem Cell Transplantation. Bone Marrow Transplant..

[102] Wang L., Allen J., Diong C., Goh Y.T., Gopalakrishnan S., Ho A., Hwang W., Lim F., Oon L., Tan T.T. (2017). Respiratory Virus Infection after Allogeneic Hematopoietic Stem Cell Transplant in a Tropical Center: Predictive Value of the Immunodeficiency Scoring Index. Transpl. Infect. Dis..

[103] Yue C., Kang Z., Ai K., Xu D., Wu J., Pan Y., Yan J., Liu M., Liu Q. (2016). Virus Infection Facilitates the Development of Severe Pneumonia in Transplant Patients with Hematologic Malignancies. Oncotarget.

[104] Mccoy D., Wong E., Kuyumjian A.G., Wynd M.A., Sebti R., Munk G.B. (2011). Treatment of Respiratory Syncytial Virus Infection in Adult Patients with Hematologic Malignancies Based on an Institution-Specific Guideline. Transpl. Infect. Dis..

[105] Lee I., Barton T.D. (2007). Viral Respiratory Tract Infections in Transplant Patients Epidemiology, Recognition and Management. Drugs.

[106] Bylsma L.C., Suh M., Movva N., Fryzek J.P., Nelson C.B. (2022). Mortality among US Infants and Children Under 5 Years of Age with Respiratory Syncytial Virus and Bronchiolitis: A Systematic Literature Review. J. Infect. Dis..

[107] Celante H., Oubaya N., Fourati S., Beaune S., Khellaf M., Casalino E., Ricard J.D., Vieillard-Baron A., Heming N., Mekontso Dessap A. (2023). Prognosis of Hospitalised Adult Patients with Respiratory Syncytial Virus Infection: A Multicentre Retrospective Cohort Study. Clin. Microbiol. Infect..

[108] Whimbey E., Champlin R.E., Couch R.B., Englund J.A., Goodrich J.M., Raad I., Przepiorka D., Lewis V.A., Mirza N., Yousuf H. (1996). Community Respiratory Virus Infections among Hospitalized Adult Bone Marrow Transplant Recipients. Clin. Infect. Dis..

[109] Hertz M.I., Englung J.A., Snover D., Bitterman P.B., McGlave P.B. (1989). Respiratory Syncytial Virus-Induced Acute Lung Injury in Adult Patients with Bone Marrow Transplant: A Clinical Approach and Review of the Literature. Medicine.

[110] Waghmare A., Campbell A.P., Xie H., Seo S., Kuypers J., Leisenring W., Jerome K.R., Englund J.A., Boeckh M. (2013). Respiratory Syncytial Virus Lower Respiratory Disease in Hematopoietic Cell Transplant Recipients: Viral RNA Detection in Blood, Antiviral Treatment, and Clinical Outcomes. Clin. Infect. Dis..

[111] Chemaly R.F., Ghosh S., Bodey G.P., Rohatgi N., Safdar A., Keating M.J., Champlin R.E., Aguilera E.A., Tarrand J.J., Raad I.I. (2006). Respiratory Viral Infections in Adults with Hematologic Malignancies and Human Stem Cell Transplantation Recipients: A Retrospective Study at a Major Cancer Center. Medicine.

[112] Ljungman P. (2014). Respiratory Syncytial Virus in Hematopoietic Cell Transplant Recipients: Factors Determining Progression to Lower Respiratory Tract Disease. J. Infect. Dis..

[113] Shah D.P., Ghantoji S.S., Mulanovich V.E., Ariza-Heredia E.J., Chemaly R.F. (2012). Review Article Management of Respiratory Viral Infections in Hematopoietic Cell Transplant Recipients. Am. J. Blood Res..

[114] Fontana L., Strasfeld L. (2019). Respiratory Virus Infections of the Stem Cell Transplant Recipient and the Hematologic Malignancy Patient. Infect. Dis. Clin. N. Am..

[115] Manuel O., Estabrook M. (2019). RNA Respiratory Viral Infections in Solid Organ Transplant Recipients: Guidelines from the American Society of Transplantation Infectious Diseases Community of Practice. Clin. Transplant..

[116] Loubet P., Lenzi N., Valette M., Foulongne V., Krivine A., Houhou N., Lagathu G., Rogez S., Alain S., Duval X. (2017). Clinical Characteristics and Outcome of Respiratory Syncytial Virus Infection among Adults Hospitalized with Influenza-like Illness in France. Clin. Microbiol. Infect..

[117] Paulsen G.C., Danziger-Isakov L. (2017). Respiratory Viral Infections in Solid Organ and Hematopoietic Stem Cell Transplantation. Clin. Chest Med..

[118] Kim Y.J., Guthrie K.A., Waghmare A., Walsh E.E., Falsey A.R., Kuypers J., Cent A., Englund J.A., Boeckh M. (2014). Respiratory Syncytial Virus in Hematopoietic Cell Transplant Recipients: Factors Determining Progression to Lower Respiratory Tract Disease. J. Infect. Dis..

[119] Harrington R.D., Hooton T.M., Hackman R.C., Storch G.A., Osborne B., Gleaves C.A., Benson A., Meyers J.D. (1992). An Outbreak of Respiratory Syncytial Virus in a Bone Marrow Transplant Center. J. Infect. Dis..

[120] Kelly S.G., Metzger K., Bolon M.K., Silkaitis C., Mielnicki M., Cullen J., Rooney M., Blanke T., Tahboub A.E., Noskin G.A. (2016). Respiratory Syncytial Virus Outbreak on an Adult Stem Cell Transplant Unit. Am. J. Infect. Control.

[121] Mendes E.T., Ramos J., Peixoto D., Dulley F., Alves T., Vilas Boas L.S., Batista M.V., Da Silva D.P., Levin A.S., Shikanai-Yasuda M.A. (2013). An Outbreak of Respiratory Syncytial Virus Infection in Hematopoietic Stem Cell Transplantation Outpatients: Good Outcome without Specific Antiviral Treatment. Transpl. Infect. Dis..

[122] Kassis C., Champlin R.E., Hachem R.Y., Hosing C., Tarrand J.J., Perego C.A., Neumann J.L., Raad I.I., Chemaly R.F. (2010). Detection and Control of a Nosocomial Respiratory Syncytial Virus Outbreak in a Stem Cell Transplantation Unit: The Role of Palivizumab. Biol. Blood Marrow Transplant..

[123] Lehners N., Tabatabai J., Prifert C., Wedde M., Puthenparambil J., Weissbrich B., Biere B., Schweiger B., Egerer G., Schnitzler P. (2016). Long-Term Shedding of Influenza Virus, Parainfluenza Virus, Respiratory Syncytial Virus and Nosocomial Epidemiology in Patients with Hematological Disorders. PLoS ONE.

[124] Weigt S.S., Gregson A.L., Deng J.C., Lynch J.P., Belperio J.A. (2011). Respiratory Viral Infections in Hematopoietic Stem Cell and Solid Organ Transplant Recipients. Semin. Respir. Crit. Care Med..

[125] Riccò M., Corrado S., Palmieri S., Marchesi F. (2023). Respiratory Syncytial Virus: A Systematic Review and Meta-Analysis of Tomographic Findings (2000–2022). Children.

[126] Auvinen R., Syrjänen R., Ollgren J., Nohynek H., Skogberg K. (2022). Clinical Characteristics and Population-Based Attack Rates of Respiratory Syncytial Virus versus Influenza Hospitalizations among Adults—An Observational Study. Influenza Other Respir. Viruses.

[127] Baraldi E., Checcucci Lisi G., Costantino C., Heinrichs J.H., Manzoni P., Riccò M., Roberts M., Vassilouthis N. (2022). RSV Disease in Infants and Young Children: Can We See a Brighter Future?. Hum. Vaccin. Immunother..

[128] Riccò M., Ferraro P., Peruzzi S., Zaniboni A., Ranzieri S. (2022). Respiratory Syncytial Virus: Knowledge, Attitudes and Beliefs of General Practitioners from North-Eastern Italy (2021). Pediatr. Rep..

[129] Hall C.B. (2001). Respiratory Syncytial Virus and Parainfluenza Virus. N. Engl. J. Med..

[130] Hall C.B., Long C.E., Schnabel K.C. (2001). Respiratory Syncytial Virus Infections in Previously Healthy Working Adults. Clin. Infect. Dis..

[131] Simpson E., Dazzi F. (2019). Bone Marrow Transplantation 1957–2019. Front. Immunol..

[132] Granot N., Storb R. (2020). History of Hematopoietic Cell Transplantation: Challenges and Progress. Haematologica.

[133] Karhana S., Hussain K., Bint-E-attar G., Bhurani D., Khan M.A. (2023). Risk of Mortality in Bone Marrow Transplant Patients During SARS-CoV-2 Infection: A Systematic Review. Exp. Clin. Transplant..

[134] Müller O., Razum O., Jahn A. (2021). Effects of Non-Pharmaceutical Interventions against COVID-19 on the Incidence of Other Diseases. Lancet Reg. Health-Eur..

[135] Flaxman S., Mishra S., Gandy A., Unwin H.J.T., Mellan T.A., Coupland H., Whittaker C., Zhu H., Berah T., Eaton J.W. (2020). Estimating the Effects of Non-Pharmaceutical Interventions on COVID-19 in Europe. Nature.

[136] Riccò M., Peruzzi S., Balzarini F. (2021). Public Perceptions on Non-Pharmaceutical Interventions for West Nile Virus Infections: A Survey from an Endemic Area in Northern Italy. Trop. Med. Infect. Dis..

[137] Riccò M., Ferraro P., Peruzzi S., Zaniboni A., Satta E., Ranzieri S. (2022). Excess Mortality on Italian Small Islands during the SARS-CoV-2 Pandemic: An Ecological Study. Infect. Dis. Rep..

[138] Weiner J.H. (2010). Respiratory Syncytial Virus Infection and Palivizumab: Are Families Receiving Accurate Information?. Am. J. Perinatol..

[139] Mitchell I., Li A., Bjornson C.L., Lanctot K.L., Paes B.A. (2022). Respiratory Syncytial Virus Immunoprophylaxis with Palivizumab: 12-Year Observational Study of Usage and Outcomes in Canada. Am. J. Perinatol..

[140] Frogel M.P., Stewart D.L., Hoopes M., Fernandes A.W., Mahadevia P.J. (2010). A Systematic Review of Compliance with Palivizumab Administration for RSV Immunoprophylaxis. J. Manag. Care Pharm..

[141] Boeckh M., Berrey M.M., Bowden R.A., Crawford S.W., Balsley J., Corey L. (1998). Phase 1 Evaluation of the Respiratory Syncytial Virus-Specific Monoclonal Antibody Palivizumab in Recipients of Hematopoietic Stem Cell Transplants Downloaded From. J. Infect. Dis..

[142] Sanders S.L., Agwan S., Hassan M., van Driel M.L., Del Mar C.B. (2019). Immunoglobulin Treatment for Hospitalised Infants and Young Children with Respiratory Syncytial Virus Infection. Cochrane Database Syst. Rev..

[143] Olchanski N., Hansen R.N., Pope E., D’Cruz B., Fergie J., Goldstein M., Krilov L.R., McLaurin K.K., Nabrit-Stephens B., Oster G. (2018). Palivizumab Prophylaxis for Respiratory Syncytial Virus: Examining the Evidence around Value. Open Forum Infect. Dis..

[144] Sánchez Luna M., Manzoni P., Paes B., Baraldi E., Cossey V., Kugelman A., Chawla R., Dotta A., Rodríguez Fernández R., Resch B. (2020). Expert Consensus on Palivizumab Use for Respiratory Syncytial Virus in Developed Countries. Paediatr. Respir. Rev..

[145] Mac S., Sumner A., Duchesne-Belanger S. (2019). Cost-Effectiveness of Palivizumab for Respiratory Syncytial Virus: A Systematic Review. Pediatrics.

[146] Esposito S., Abu Raya B., Baraldi E., Flanagan K., Martinon Torres F., Tsolia M., Zielen S. (2022). RSV Prevention in All Infants: Which Is the Most Preferable Strategy?. Front. Immunol..

[147] Voirin N., Virlogeux V., Demont C., Kieffer A. (2021). Potential Impact of Nirsevimab on RSV Transmission and Medically Attended Lower Respiratory Tract Illness Caused by RSV: A Disease Transmission Model. Infect. Dis. Ther..

[148] Jones J.M., Fleming-Dutra K.E., Prill M.M., Roper L.E., Brooks O., Sánchez P.J., Kotton C.N., Mahon B.E., Meyer S., Long S.S. (2023). Use of Nirsevimab for the Prevention of Respiratory Syncytial Virus Disease among Infants and Young Children: Recommendations of the Advisory Committee on Immunization Practices-United States, 2023. Morb. Mortal. Wkly. Rep..

[149] Francisco L., Cruz-Cañete M., Pérez C., Couceiro J.A., Otheo E., Launes C., Rodrigo C., Jiménez A.B., Llorente M., Montesdeoca A. (2023). Nirsevimab for the Prevention of Respiratory Syncytial Virus Disease in Children: Statement of the Spanish Society of Paediatric Infectious Disease (SEIP). An. Pediatría (Engl. Ed.).

[150] Domachowske J.B., Anderson E.J., Goldstein M. (2021). The Future of Respiratory Syncytial Virus Disease Prevention and Treatment. Infect. Dis. Ther..

[151] Azzari C., Baraldi E., Bonanni P., Bozzola E., Coscia A., Lanari M., Manzoni P., Mazzone T., Sandri F., Checcucci Lisi G. (2021). Epidemiology and Prevention of Respiratory Syncytial Virus Infections in Children in Italy. Ital. J. Pediatr..

[152] Verwey C., Madhi S.A. (2023). Review and Update of Active and Passive Immunization Against Respiratory Syncytial Virus. BioDrugs.

[153] Bouzid D., Visseaux B., Ferré V.M., Peiffer-Smadja N., Le Hingrat Q., Loubet P. (2023). Respiratory Syncytial Virus in Adults with Comorbidities: An Update on Epidemiology, Vaccines, and Treatments. Clin. Microbiol. Infect..

[154] Cunningham C.K., Karron R.A., Muresan P., Kelly M.S., McFarland E.J., Perlowski C., Libous J., Oliva J., Jean-Philippe P., Moye J. (2022). Evaluation of Recombinant Live-Attenuated Respiratory Syncytial Virus (RSV) Vaccines RSV/ΔNS2/Δ1313/I1314L and RSV/276 in RSV-Seronegative Children. J. Infect. Dis..

[155] Vidal Valero M. (2023). “A Good Day”: FDA Approves World’s First RSV Vaccine. Nature.

[156] Miller P.D.E., Patel S.R., Skinner R., Dignan F., Richter A., Jeffery K., Khan A., Heath P.T., Clark A., Orchard K. (2023). Joint Consensus Statement on the Vaccination of Adult and Paediatric Haematopoietic Stem Cell Transplant Recipients: Prepared on Behalf of the British Society of Blood and Marrow Transplantation and Cellular Therapy (BSBMTCT), the Children’s Cancer and Leukaemia Group (CCLG), and British Infection Association (BIA). J. Infect..

